# Snake Toxins Affecting Blood Vessel Walls: Mode of Action and Biological Significance

**DOI:** 10.3390/ijms26199439

**Published:** 2025-09-26

**Authors:** Alexey V. Osipov, Yuri N. Utkin

**Affiliations:** Shemyakin-Ovchinnikov Institute of Bioorganic Chemistry, Russian Academy of Sciences, Moscow 117997, Russia; osipov-av@ya.ru

**Keywords:** angiogenesis, blood vessel, capillary permeability, cell adhesion, integrin, snake, toxin, vascular tone, vasorelaxation, venom

## Abstract

One of the main targets for snake venoms in animal and human organisms is the circulatory system. Mechanisms of circulatory system injury within the victim’s body include, among others, the direct effect of snake toxins on structures in blood vessel walls. The interaction of a toxin with cells and the extracellular matrix of the vessel wall may manifest as cytotoxicity, leading to cell death by necrosis or apoptosis, and damage to vascular wall structures. Such interactions may increase capillary permeability, promoting hemorrhage or edema, and may also induce alterations in vascular tone, resulting in changes in blood pressure. Snake toxins may also affect the growth, function, and regenerative ability of the endothelium, thus modulating angiogenesis; some toxins exert protective or anti-atherosclerotic effects. Toxins interacting with the vasculature may be classified as enzymes (phospholipases A_2_, metalloproteinases, L-amino acid oxidases, and hyaluronidases), proteins without enzymatic activity (vascular endothelial growth factors, disintegrins, C-type lectins and snaclecs, three-finger toxins, etc.), peptides (bradykinin-potentiating peptides, natriuretic peptides, sarafotoxins), and low-molecular-weight substances. This review summarizes the data on the vascular effects, particularly on the blood vessel wall, exhibited by various classes and groups of snake toxins.

## 1. Introduction

Snake venom is a concentrated solution of compounds, called toxins, with high biological activity and selectivity toward targets within their victim organism. As a rule, about 90% (or more) of the dry weight of snake venom consists of toxins of a protein/peptide nature, which may be either enzymes or toxins without enzymatic activity. To act most effectively, toxins target the organism’s major vital systems, which include the nervous and cardiovascular (CVS) systems.

### 1.1. Snake Venom Damaging Strategies Within the CVS

Snake toxins can affect the CVS in four main ways:

- By interfering with blood clotting. The toxins can affect both plasma hemostasis and platelet aggregation, acting as either anticoagulants or prothrombotics [1,2];
- By having a systemic or direct effect on the myocardium and the heart as a whole [3,4];
- By acting systemically on the tone of the vascular wall. For example, inhibition of the angiotensin-converting enzyme in the lung or kidney by toxins results in vessel relaxation throughout the bloodstream [5];
- By acting directly on the walls of blood vessels.

The first three ways were reviewed recently (see references [2,3,4,5]), while the data about direct effects of snake toxins on the blood vessels have not been systematized. The aim of this review is to consider the fourth way. The direct effects of snake toxins on the condition of vascular walls are complex and varied and obviously have both fundamental and applied significance. Blood vessels are the tubular structures of the CVS that transport blood throughout animal bodies. Blood vessels can be classified into five main types: arteries, which transport blood away from the heart; arterioles; capillaries, where the exchange of water and chemicals between the blood and tissues occur; venules; and veins, which transport blood back to the heart. It should be noted that the heart and blood vessels, although forming an entire CVS and having a common mesodermal origin, differ significantly in their structure, functions and innervation, so pharmacological agents, in some cases, can affect them in opposite ways. However, the main blood vessels—arteries and veins—in general, have a similar wall structure, although some differences exist between them. So, in general, the inner layer in arteries and veins is the endothelium on the basement membrane (BM), the middle layer contains smooth muscle fibers, and the outer layer is a structural support of the vessel. It should be mentioned that capillaries have only endothelium, but the endothelium is the main target for snake toxins; that will be discussed below. This review summarizes information on the direct effects of toxins in relation to the walls of blood vessels. Only those papers are considered where it is clearly shown that the targets for snake toxins are located in at least one of the layers of the vessel wall.

**Figure 1:**
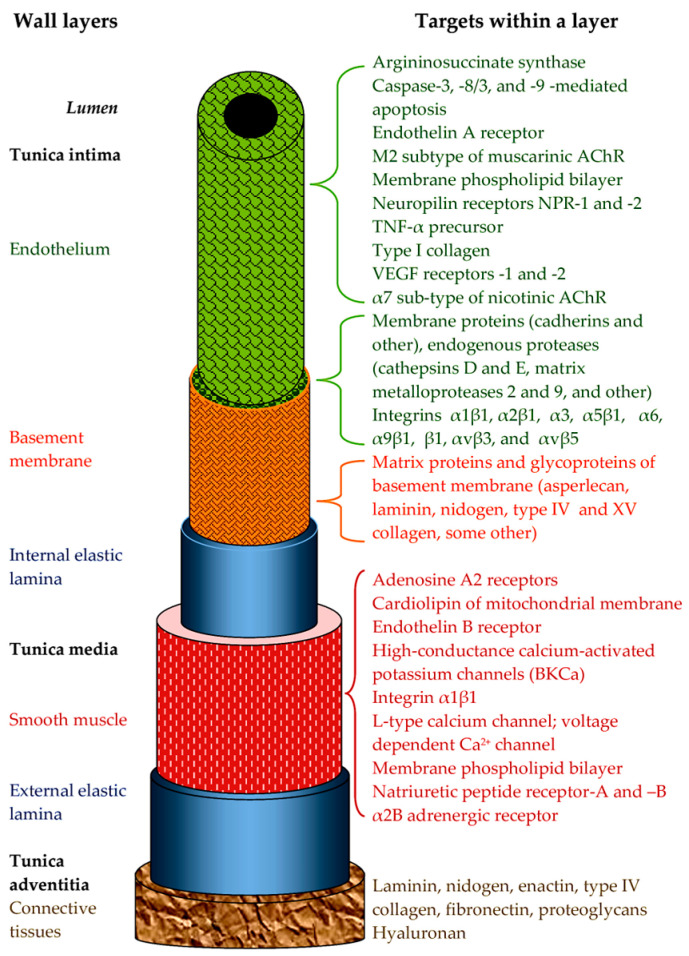
Schematic representation of a blood vessel listing established targets for snake venom toxins within the layers of a vessel wall (considered in detail in Section 2, Section 3 and Section 4).

Snake venom toxins can affect all three layers of the blood vessel walls (Figure 1). Their effects can be described in the following ways:

(i) The pharmacological activity of toxins aimed at changing vascular tone. Here, it is necessary to distinguish between direct effects on the cells of the vessel wall and indirect effects through nervous or humoral regulation. In this paper, we will only consider direct effects.
(ii) The effects on the regenerative ability of vessel tissues. This includes effects on the growth and functioning of the vascular epithelium, as well as on neoangiogenesis.
(iii) Direct damage to the structures of the vascular wall. Snake toxins can damage the plasma membranes of cells, disrupt intercellular interactions, and cleave the components of the extracellular matrix.
(iv) Snake toxins, in addition to having damaging effects, can also have a protective effect on blood vessels.

The consideration of toxin effects will follow this order. It should be noted that toxins belonging to the same class, or even the same toxin, can often exhibit several effects simultaneously. In some instances, a single trigger toxin effect can initiate a whole series of other parallel effects; in other cases, the development of the first effect provokes the emergence of a cascade of subsequent ones; however, in certain cases, the relation between the various effects of the toxin is not so obvious. These facts raise the question of the specificity of the toxins’ action. As an example, let us consider cobra cytotoxins. These toxins exhibit rather non-specific cytotoxicity; however, they show some preference for cardiomyocytes as well as dividing cells. Therefore, most of the work on these compounds has been carried out on cardiomyocytes and tumor cells, perhaps for utilitarian reasons. Certainly, the known effects of cytotoxins can be extrapolated with some approximation to the cells of the vascular wall. However, such an extrapolation would not be entirely correct, since, as has been shown previously [6], the same cytotoxin may show different effects on the heart muscle and the aortic muscle, especially at different concentrations, within a single experiment. The same considerations can be applied to other snake toxins. Therefore, this review will be limited to a discussion of work performed specifically on blood vessel walls or related cells.

### 1.2. Snake Venom Toxins Affecting the CVS

To date, snake toxins of the following classes have been reported to affect the CVS:

*Toxins possessing enzymatic activity*. These include metalloproteinases (SVMPs), serine proteinases, phospholipases A_2_ (PLA_2_s), hyaluronidase, L-amino acid oxidases (LAAOs), and 5-nucleotidase. Their effect is often (but not always) directly related to their enzymatic activity. However, in some cases, there is no relation between their enzymatic activity and their biological effects.

*Toxins without enzymatic activity*. This group is very diverse and comprises both proteins and peptides. Proteinaceous toxins are C-type lectins and C-type lectin-like proteins, vascular endothelial growth factors (VEGFs) and nerve growth factors (NGFs), cysteine-rich secretory proteins (CRISPs), three-finger toxins (cytotoxins, α-neurotoxins, calciseptine), disintegrins, and Kunitz-type inhibitors. Of the peptide toxins, bradykinin-potentiating peptides (BPPs), natriuretic peptides (NPs), and sarafotoxins should be mentioned. Moreover, low-molecular-weight organics—for example, adenosine—may also affect the CVS. The effects of these toxins are mediated by their binding to the corresponding receptors, ion channels, and other molecular targets.

It should be kept in mind that the genes encoding snake venom toxins have an increased tendency to duplicate and mutate. Therefore, toxins are often present in the venom of one snake species, and even in one specimen, as a pool of several homologs; these might only differ slightly from each other in structure but can greatly differ in their biological activity, sometimes even acting in a completely opposite manner. In order to avoid confusion when discussing the effects of a particular snake toxin of any class, both its source (snake species) and its name, if given by the authors, will be pointed out.

## 2. Toxins Affecting Vascular Tone

In most cases, snake venom toxins reduce vascular tone by directly acting on vessels, but such an effect can also be indirect. In this review, we do not intend to consider indirect vasodilatory effects. These effects include, but are not limited to, for example, the systemic release of histamine by mast cells under the action of PLA_2_s or SVMPs, an increase in the concentration of bradykinin in the bloodstream under the action of viper kallikrein-like serine proteases, or the blocking of angiotensin-converting enzyme 1 (ACE1) with BPPs, etc. These are considered in a good mini-review on snake toxins exerting hypotensive effect(s) [5]. The snake toxins directly affecting vascular tone are discussed in this chapter.

### 2.1. Bradykinin-Potentiating Peptides

**Figure 2:**
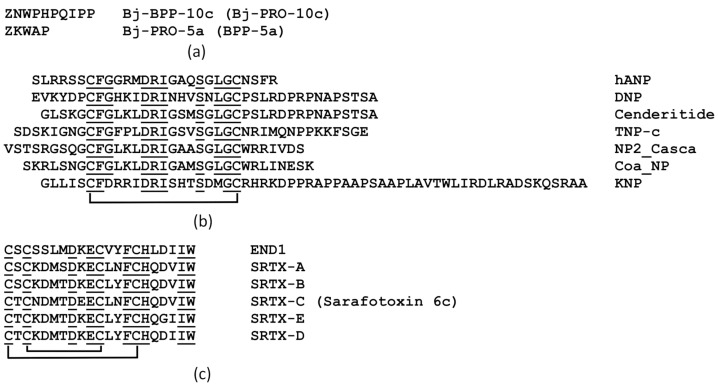
Amino acid sequences of BPPs (**a**), NPs (**b**) and safafotoxins (**c**). Z = pyroGlu. Bj-BPP-10c (UniProt accession ID Q6LEM5) and Bj-PRO-5a (Q6LEM5) are from *Bothrops jararaca*. hANP, human NP of A-type; DNP, NP from *Dendroaspis angusticeps* (P28374); TNP-c, NP from *Oxyranus microlepidotus* (P83230); KNP, NP from *Bungarus flaviceps* (D5J9S0); NP2_Casca, NP from *Crotalus durissus cascavella* (P0DKY6); Coa_NP, NP from *C. oreganus abyssus* (B3EWY3). END1, human endothelin 1 (P05305); SRTX-A, SRTX-B, SRTX-C, SRTX-E, and SRTX-D represent sarafotoxins A (P13208), B (P13208), C (P13208), E (P13208), and D (P13211) from *Atractaspis engaddensis*. Disulfide bonds are shown as lines connecting cysteine residues. Invariant residues within each group are underlined.

BPPs found in the venom gland of the *Bothrops* pit vipers are proline-rich oligopeptides composed typically of 5 to 14 amino acid residues, with a pyroglutamate and a proline residue at their N- and C-termini, respectively (Figure 2a). These peptides are bradykinin-degradation inhibitors as well as the first naturally occurring inhibitors of ACE1 to be described.

Along with the generalized antihypertensive effect mediated through the renin–angiotensin system, BPPs from pit viper venom also have a local vasodilating effect. So, the BPP Bj-BPP-10c from *Bothrops jararaca* manifests a strong, sustained antihypertensive effect, independent of ACE1 inhibition, in spontaneous hypertensive rats without causing any effect in normotensive rats. When applied to HEK293 cells, Bj-PRO-10c recognizes argininosuccinate synthase (AsS), a key enzyme that provides substrates for NO-synthase (NOS), as a main target and activates it dose-dependently [7]. Another BPP from the same snake, Bj-PRO-5a, induces dose-dependent NO production in HEK293 cells, where it seems to interact with the muscarinic acetylcholine receptor M1 and bradykinin receptor B2 (BKB2). This results in a synergistic regulation of NO synthesis. HOE-140, an antagonist of BKB2, or pirenzepine, an inhibitor of the M1 muscarinic receptor, significantly reduces the effect of Bj-PRO-5a [8]. Although these targets for BPPs have been found in HEK293, vascular epithelial cells also possess similar pathways for the induction of NO synthesis, which is a powerful vasodilator. Indeed, fluorescently labelled Bj-PRO-10c is homogenously distributed within the cell cytoplasm in human umbilical vein endothelial cells (HUVECs) in the same manner as within HEK293 cells. The concurrent treatment of HUVECs with Bj-BPP-10c and with N(ω)-nitro-L-arginine methyl ester (L-NAME), a specific inhibitor of NOS, or with methyl-D-L-aspartic acid (MDLA), a specific inhibitor of AsS, completely abolishes the effect of Bj-BPP-10c on NO production, indicating the involvement of NOS and AsS in the observed increase of NO metabolite production by the peptide [7].

Endothelium-containing and endothelium-denuded aortic rings (ARs) of spontaneously hypertensive Wistar rats have been used to evaluate whether BPP-5a causes cardiovascular effects by a direct vasorelaxant effect. So, in ARs of spontaneously hypertensive rats, concentration-dependent vasorelaxation is produced by BPP-5a, while the absence of a functional endothelium cancels this effect. The lack of association of BPP-5a-induced vasorelaxation with the BKB2 receptor or prostaglandins has been confirmed by the use of their inhibitors, HOE 140 and indomethacin, respectively. Conversely, the NOS inhibitor, L-NAME, completely blocks the vasorelaxant effect caused by BPP-5a, indicating that the cardiovascular effect of the pentapeptide is due to increases in endothelial NO release [9]. In contrast, the vasorelaxant effect induced by BPP-5a was completely blocked by the NOS inhibitor, L-NAME, indicating that increased endothelial NO release is the primary cause of the cardiovascular pentapeptide effects.

Interactions of 11 BBPs from the pit viper *B. moojeni* with the AsS enzyme have been investigated in silico and suggest a putative binding pocket for these molecules in the AsS enzyme. A binding cavity for BPPs has been predicted, and docking studies have allowed BPPs to be ranked according to their activity. Three peptides have been synthesized, all of which showed varying degrees of activity in vasorelaxation assays using rat ARs [10].

### 2.2. Natriuretic Peptides

NPs are short-life peptide hormones with hypotensive activity associated with vasodilation, natriuresis, and diuresis. They consist of approximately 20–50 amino acid residues and include a conserved 17-amino acid sequence bounded by a disulfide bond (Figure 2b). This 17-residue ring is essential for the binding to, and for specificity toward, their receptors (NPR of -A, -B, and -C sub-types). Venoms of snakes belonging to both the Viperidae and Elapidae families may contain NPs. These peptides share the same 17-residue ring structure as mammalian NPs but differ in the length and sequence of their N-terminal and C-terminal tails, being much more resistant to endopeptidase degradation [11].

NPs, in addition to their systemic effects, have local vasodilator effects that can be realized through the following ways:

- By endothelium-dependent vasorelaxation with increased NO production;
- By lowering blood pressure by reducing vascular resistance (due to a decrease in the influx of Ca^2+^ ions into muscle cells);
- By possibly also acting through K^+^ channels [3].

The natriuretic peptide DNP identified in the venom of the green mamba *Dendroaspis angusticeps* is more resistant to peptidase degradation than other NPs. Like the mammalian NP of A-type, DNP fully reverses the constrictor response to endothelin-1 in an endothelium-intact or denuded mammary artery, with comparable nanomolar potencies [12]. The observed vasodilatation has been shown to be predominantly mediated via direct activation of the smooth muscle natriuretic peptide receptor-A (NPR-A) [12].

NPs from the inland taipan *Oxyranus microlepidotus* venom are represented by three isoforms, namely TNP-a, TNP-b, and TNP-c; the same peptides are also present in the venoms of *O. scutellatus canni* (New Guinea taipan) and *O. s. scutellatus* (coastal taipan). TNP-c is equipotent to the A-type NP in AR assays whereas TNP-a and TNP-b are either inactive or weakly active. TNP-a and TNP-b are also unable to inhibit the binding of TNP-c in endothelium-denuded aortas or endothelium-intact aortas [13].

The C-terminus of KNP, an NP from the venom of the red-headed krait *Bungarus flaviceps*, has a longer sequence of 38 amino acid residues, the last 21 residues of which form an -helix. The disulfide-confined ring in a typical NP interacts with the NPR-A receptor and activates a pathway mediated by cGMP. On the contrary, the α-helix, by untypically binding to an unknown receptor, produces NO-dependent vasodilation. Even at concentrations 100 times higher than A-NP, KNP causes endothelium-dependent vasodilation without significant renal effects (natriuresis and diuresis) [14]. The C-terminal tail of TcNP-a, an NP from the venom of the rough-scaled snake *Tropidechis carinatus*, contains O-glycosylated amino acid residue(s). Both the NPR-A and NPR-B receptors are continuously activated by both glycosylated and non-glycosylated TcNP-a [15].

A relaxant effect is produced by NP2_Casca, an NP from *Crotalus durissus cascavella* venom, in endothelium-intact thoracic ARs precontracted with phenylephrine in the presence and absence of isatin, a natriuretic receptor antagonist [16]. Coa_NP, an NP-like peptide from *C. oreganus abyssus* venom, also produces vasorelaxation in thoracic ARs precontracted with phenylephrine [17]. Both NP2_Casca and Coa_NP fail to relax ARs precontracted with an isosmotic potassium solution, which allows the authors to suggest a possible role for potassium channels [16,17].

### 2.3. Sarafotoxins

Sarafotoxins, potent vasoconstrictor peptides of 2.5 kDa that are over-produced in *Atractaspis* venoms, form a separate toxin family. They share a common structural motif comprising 21 amino acids and two invariant disulfide bonds between cysteines 1–15 and 3–11 (Figure 2c). Sarafotoxins are closely related to endothelins, which are mammalian vasoconstrictor hormones, in structure (with approximately 60% sequence identity) and function. There are two types of endothelin receptors—ETA and ETB. The former, which mediate vasoconstriction, are predominant on vascular smooth muscle cells; the latter, the endothelial ETB receptors, can mediate vasodilation via nitric oxide (NO) release and hemodilution.

Almost all sarafotoxins cause abrupt vasoconstriction; however, sarafotoxin S6c, an agonist of endothelin receptor ETB, evokes pronounced vasodilatation. S6c is used intensively in ETB-mediated vasodilatation studies for different normal and pathological conditions. Some of these investigations have shed light on the peculiarities of S6c action. For example, in isolated rings of left pulmonary arteries or thoracic aortas from healthy rats, ETB receptor stimulation with sarafotoxin S6c induces vasorelaxation, while no vasoconstriction has been observed. However, in arteries isolated from rats with systemic or pulmonary hypertension, ETB-mediated endothelial relaxation is abolished, whereas sarafotoxin S6c stimulation results in vasoconstriction. Such a distortion of the main effect can be explained by the occurrence of endothelial dysfunction, in which ETB expression in the endothelium is reduced, while ETB expression is increased in smooth muscle cells, where it mediates vasoconstriction [18].

Among the vasoconstrictor sarafotoxins, S6b has been used most intensively in cardiovascular studies. Some of these works clarify its mechanism of action. So, S6b is a strong vasoconstrictor equipotent to endothelins ET-1 and ET-2, with comparable maximum responses in human saphenous veins. In a β-arrestin assay carried out using CHO-K1 cells, however, S6b appears to be only a partial agonist when compared with ET-1 [19]. A crystal structure of the human ETB receptor in a complex with sarafotoxin S6b has been resolved; the authors discuss structural differences in S6b and S6c that might be responsible for the opposing effects of these toxins [20].

### 2.4. Three-Finger Toxins

**Figure 3:**
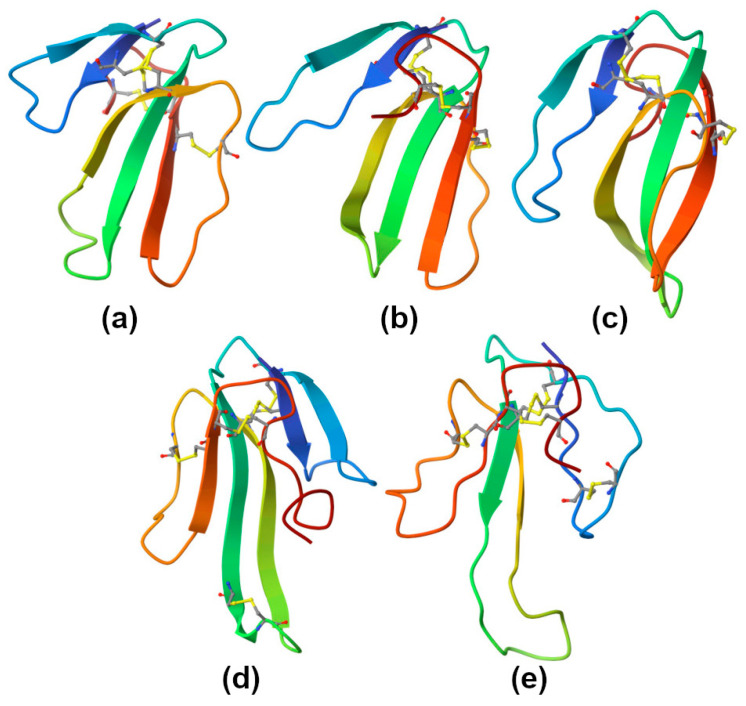
Spatial structures of TFTs. (**a**) Cytotoxin 1 from *Naja oxiana* venom (PDB 5NPN); (**b**) Muscarinic toxin MT9 from *D. polylepis* (PDB 6R5M); (**c**) Toxin FS2 from *D. polylepis* (PDB 1TFS); (**d**) α-Bungarotoxin from *Bungarus multicinctus* (PDB 1KFH); (**e**) γ-Bungarotoxin from *B. multicinctus* (PDB 1MR6). The structure of MT9 was determined by X-ray crystallography; all other structures were determined by NMR spectroscopy. Disulfide bonds are shown in yellow.

Three-finger toxins (TFTs) are prevailing components in Elapidae snake venoms. Their polypeptide chains of 60–74 amino acid residues form three β-stranded loops extending from a small, globular, hydrophobic core stabilized by four conserved disulfide bridges (Figure 3). TFTs of certain types, including long-chain α-neurotoxins, non-conventional toxins, and muscarinic toxins, contain an additional fifth disulfide, while others, such as short-chain α-neurotoxins, cytotoxins, calciseptine, and some others, do not. These toxins can recognize a broad range of molecular targets. For example, α-neurotoxins block certain sub-types of nicotinic acetylcholine receptors; calciseptine is an L-type calcium-channel blocker; cytotoxins target cell membranes. It should be noted that, being highly versatile, TFTs may recognize many other targets [21].

#### 2.4.1. Cytotoxins

The effect of cobra cytotoxins (CTs) on blood vessels leads to a two-phase change in vascular tone. In ARs with an intact endothelium, CTs first produce a transient relaxation with following sustained contraction. Preliminary incubation of ARs with L-NAME, an NOS inhibitor, or endothelium removal cancels the transient relaxation but does not influence the magnitude of the contractile response caused by CTs. CTs themselves not only produce contraction of vascular smooth muscle in a concentration-dependent manner but also diminish contractions induced by KCl stimulation or phenylephrine. CT-induced contraction depends on the concentration of external Ca^2+^ [22]. The direct lytic factor DLF, a CT homolog from the venom of the *Naja naja atra* cobra produces a contractile response in Krebs’ solution without Ca^2+^, and subsequent addition of Ca^2+^ into the bath induces a further increase in the contraction. DLF does not produce contractions after the depletion of intracellular Ca^2+^ by phenylephrine. It induces the release of Ca^2+^ from phenylephrine-sensitive intracellular Ca^2+^ stores and an influx of extracellular Ca^2+^ through voltage-gated Ca^2+^ channels [23]. On the other hand, a cardiotoxin (another name for a CT) from the venom of the same cobra species induces the contraction of endothelium-denuded rat ARs that is associated with an extracellular Ca^2+^ influx process but not with the release of Ca^2+^ from intracellular stores [24]. The authors suggest that internal calcium stores in rat aortic smooth muscle are insensitive to the cardiotoxin, so it interacts extracellularly with the plasma membrane at the level of the L-type calcium channels. An interesting note: the effects of CTs on both vascular smooth muscle and endothelial cells are inhibited by high calcium concentrations [24,25]. Unfortunately, these papers do not allow us to determine whether DLF and the cardiotoxin are the same CT or if they are two distinct CTs.

CTX-1 (S-type) from *N. oxiana* cobra venom is less active than CTX-2 (P-type) as an inhibitor of contraction of ARs. Overall, this is consistent with data obtained on myocardium and some other preparations. The use of various inhibitors of Ca^2+^ exchangers and channels has shown that in the AR, different molecular mechanisms that increase the concentration of intracellular Ca^2+^ may be involved in the CT effects. Thus, nifedipine, an L-type Ca^2+^-channel blocker, and KB-R7943, a reverse-mode Na^+^/Ca^2+^ exchange inhibitor, significantly reduce CT-induced AR contraction [6].

#### 2.4.2. Muscarinic Toxins

TFTs include several types of toxins, including the so-called muscarinic toxins, which target muscarinic acetylcholine receptors and some other G-protein coupled receptors, e.g., adrenoreceptors. Muscarinic toxin α from *Dendroaspis polylepis* black mamba venom is a potent antagonist of the α2B adrenergic receptor located directly on the surface of arterial smooth muscle cells, and they can therefore be considered as a treatment for arterial pressure disorders [26].

MT9 is another muscarinic toxin from the black mamba venom, which, unlike classical snake muscarinic toxins, is a selective blocker of the M2 subtype of mAChR. It antagonizes the M2R/Gi pathways in cell-based assays. MT9 acts as a non-competitive antagonist against acetylcholine or arecaine (agonist of M1 and M2 mAChR) with low nanomolar potency, reversing fully the ex vivo activation of isolated rat mesenteric arteries and human internal mammary arteries [27].

#### 2.4.3. Calciseptine and FS2

Calciseptine and FS2 are TFTs from black mamba *D. polylepis* venom that, like nifedipine, specifically inhibit L-type Ca^2+^ channels. Blockers of these channels prevent Ca^2+^ ions from entering the cytoplasm and mediating various responses, including vasoconstriction. A recent work defined Cav1.2 (alpha1C) sub-type of L-type channels as a selective target for calciseptine [28]. Calciseptine blocks spontaneous portal vein contractions and K^+^-induced contractions of aortic smooth muscle. The blockage of portal vein contractions cannot be reversed after a 15 min washing. Upon extracellular application of 1 µM calciseptine, the action-potential amplitude and activity of aortic smooth muscle cells from the A7r5 cell line decreased rapidly but reversibly [29].

Calciseptine and FS2 produce dose-dependent relaxation in preparations of pulmonary arteries as well as rat aortas under K^+^-preconstriction, including that provoked by Bay K 8644, an L-type channel agonist. Calciseptine and FS2 cause a sustained decrease in blood pressure in vivo, with the biphasic effect including a short-lasting phase followed by a long-lasting one, in contrast to only short-lasting effects of nifedipine [30].

### 2.5. Vascular Endothelial Growth Factors

VEGFs from snake venom (F-type) resemble endogenous vertebrate VEGFs of types A–D. They act primarily through specific receptor tyrosine kinases, VEGFR-1 and 2, which are involved in angiogenesis and vascular permeability regulation. In addition, VEGFR-1 is responsible for cell migration and proliferation, whereas VEGFR-2 promotes mitogenesis [31]. There are other molecules, which VEGF can bind to, that have no catalytic function: for example, the co-receptors neuropilins NP-1 and NP-2 as well as heparin.

VEGF-Fs are heparin-binding homodimeric proteins of about 25 kDa; their amino acid sequences have about 50% identity with mammalian VEGF-A_165_ and a noticeably truncated C-terminal region. In terms of molecular structure, all VEGFs comprise a VEGF homology domain that contains 92–96 amino acid residues that are 29–64% identical within the family, and N- and C-terminal extensions. Eight cysteine residues are strictly invariant across members of the family and form a cysteine knot. Tissue-type VEGFs are highly conserved among all vertebrates, while snake venom VEGFs are highly diverse, especially in the functionally important regions, i.e., around receptor-binding loops and the C-terminal heparin and neuropilin co-receptor-binding sites [31,32]. Functionally characterized VEGF-Fs have been purified from Viperidae venoms; in Elapidae venoms, they have been identified only by omics techniques. It is known that human VEGF induces endothelium-dependent hypotension and that VEGF stimulates NO production in vascular endothelial cells.

The first VEGF-like protein has been identified in *Vipera aspis* venom; it exerts a strong hypotensive effect in vivo. It also has a mitogenic effect on endothelial cells, but its mitogenic activity is 5–10 times lower than that of mammalian VEGF [33]. Two other snake venom VEGFs with similar properties are vammin from the venom of *V. ammodytes ammodytes* and VR-1 from *Daboia russelli russelli*. Snake venom VEGFs exhibit highly specific binding to VEGFR-2, with essentially an equal affinity for human VEGF, but do not bind to other VEGF receptors at all. The binding of venom VEGFs to VEGFR-2 alone results in potent biological effects. Vammin exposure stimulates endothelial NOS phosphorylation at serine 1177, which results in enzyme overactivation. Thus, snake venom VEGFs induce nitric oxide production via activation of NOS by phosphorylation of serine 1177, similar to mammalian VEGF [34].

### 2.6. Phospholipases A_2_

**Figure 4:**
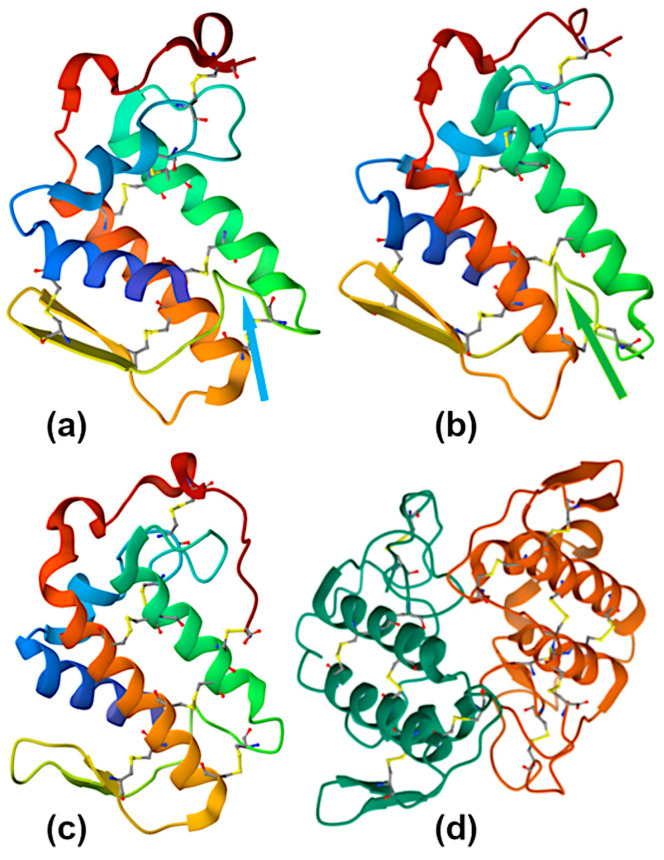
Spatial structures of some PLA_2_s as determined by X-ray crystallography. (**a**) PLA_2_ of group IA from *Naja naja* (PDB 1PSH); (**b**) PLA_2_ MiPLA2 of group IB from *Micropechis ikaheka* (PDB 1PWO); (**c**) PLA_2_ BthTx-II of group IIA from *Bothrops jararacussu* (PDB 3JR8); (**d**) Heterodimeric PLA_2_ daboiatoxin from *Daboia russelli* (PDB 2H4C). Disulfide bonds are shown in yellow. The blue arrow in (**a**) indicates the elapid loop; the green arrow in (**b**) indicates the pancreatic loop.

PLA_2_ (EC 3.1.1.4 [35]) is the most abundant protein type across overall snake proteome. Snake venom PLA_2_s are small secretory proteins with a molecular weight of 14–18 kDa, comprising 120–135 amino acid residues and 6–7 disulfide bonds stabilizing their molecular structure (Figure 4). These Ca^2+^-dependent esterases with a pH optimum of about 7 are characterized by a His48/Asp49 dyad at the active site and the presence of Ca^2+^-binding loop. Depending on their disulfide pattern, three-dimensional structure, and amino acid sequence, all secretory PLA_2_s are divided into several groups. Snake venom PLA_2_s belong to five sub-groups within two groups—IA, IB, IIA, IIB, and IIE [36].

PLA_2_s of sub-group IA are monomers ubiquitous in the venom of elapid snakes. They have seven disulfide bridges and an insertion of 2–3 amino acid residues (so-called “elapid loop”) and often exert high enzymatic activity. PLA_2_s from sub-group IB possess an additional loop of five amino acid residues, the so-called pancreatic loop. They are more common in mammals but very rare in snake venom. PLA_2_s from sub-group IIA are ubiquitous in the venom of viperid snakes. They have seven disulfides and an extension of 5–7 amino acid residues at the C-terminal region, and they often exist as non-covalent homo- or heterodimers. PLA_2_s of sub-group IIB have six disulfides and are found rarely in the venom of viperid. PLA_2_s from sub-group IIE are found in rear-fanged snakes and have been poorly studied.

PLA_2_s are hydrolases that catalyze the cleavage of glycerophospholipids at the sn-2 position. When PLA_2_ acts on cells, the main result of the reaction is the disruption of the integrity of the cell membrane and its permeabilization, leading to an uncontrolled influx of extracellular contents, including Ca^2+^ ions, into the cell, which triggers molecular mechanisms that cause cell death. Moreover, as a result of hydrolysis, arachidonic acid and lysophospholipids, being the precursors of a number of signaling molecules, are released [36]. To date, there are several proposed mechanisms of the PLA_2_ enzymatic action, which have been considered in detail in a recent review [36].

Some PLA_2_-like proteins found in snake venoms have a Lys, or Ser, or, more rarely, an Asn or Gln residue instead Asp49. These proteins have no detectable enzymatic activity. Nevertheless, they exert a broad spectrum of biological activities that are apparently not related to phospholipolysis. This can be explained by the existence of so-called “pharmacological site(s)” distinct from the enzymatic one, a concept proposed by Kini and Evans [37].

Snake venom PLA_2_s may exert either a vasorelaxant or vasoconstrictor effect. The former is much more common and pronounced. For example, the venoms of five Australian brown snakes produce a vasorelaxant effect on arteries dissected from the rat mesenteric vasculature. At least half of the effects of whole venoms are abolished by pretreatment with the specific PLA_2_ inhibitor varespladib, which means that toxins with phospholipase activity are responsible for the vasorelaxant effect [38]. These proteins appear to reduce blood pressure by locally releasing arachidonic acid, which is further metabolized by cyclooxygenases to prostaglandins or prostacyclin. ARs precontracted by norepinephrine are relaxed by PLA_2_ in a dose-dependent manner, and this effect is not reduced by endothelial ablation. Nordihydroguaiaretic acid, a lipoxygenase inhibitor, partially reduces the relaxation, while indomethacin, a cyclooxygenase inhibitor, potentiates the relaxation [39]. In mesenteric arteries with an intact endothelium, a relaxant effect was induced by two PLA_2_s from *O. scutellatus* venom. Interestingly, the effect of one PLA_2_ is significantly reduced while the relaxation produced by another PLA_2_ is practically not affected in arteries without an endothelium. Cyclooxygenase metabolites, H1-receptors, and possibly bradykinin mediate the observed antihypertensive activity [40]. In rat ARs preconstricted with 10 μM phenylephrine, a vasorelaxant effect is induced by HDP-1, a heterodimeric PLA_2_ from *V. nikolskii* venom [41]. Along this line, crotoxin, a heterodimeric neurotoxic PLA_2_ from *C. durissus terrificusis* venom, downregulates the LPS-induced production of the vasoactive factors endothelin-1 and prostaglandin I2 in HUVEC cells, suggesting a vasodilating effect [42].

In contrast, vasoconstrictive effects can be exerted by some snake venom PLA_2_s. Thus, in mouse thoracic ARs preconstricted by a high concentration of potassium chloride (60 mM), a further contractile response of about 20% is induced by AhV_aPA, a PLA_2_ from the venom of *Agkistrodon halys pallas* [43]. This vasoconstrictive effect is not significantly reduced by treatment of AhV_aPA with p-bromophenacyl bromide, a specific PLA_2_ inhibitor. This means that phospholipolytic activity is not involved in the contractile response. It has been suggested that Ca^2+^ released from the sarcoplasmic reticulum, which is related to the activation of inositol trisphosphate receptors, could produce this effect [44]. In rat ARs both with and without an endothelium, contractions are induced by crotoxin. This crotoxin effect is blocked by indomethacin, but not by L-NAME. It has been suggested that the observed contractile response is dependent on the activation of cyclooxygenase by crotoxin [45].

Whole venoms of brown Australian snakes *Pseudonaja affinis* and *P. textilis* markedly inhibit sympathetic nerve-mediated vascular contractions of isolated mesenteric arteries. As pretreatment with varespladib, a PLA_2_ inhibitor, significantly attenuates this sympatholytic effect, it is reasonable to assume that some forms of venom PLA_2_s act at synaptic terminals in vascular smooth muscle; alternatively, they might act post-synaptically at the α1-adrenoceptor (although PLA_2_s have not been reported to affect the function of α1-adrenoceptors in vascular smooth muscle) [38].

### 2.7. Cysteine-Rich Secretory Proteins

CRISPs possess a single polypeptide chain composed of about 220 amino acid residues with eight conserved disulfide bonds. CRISPs have two main domains, a CAP/PR-1 domain at the N-terminus and a cysteine-rich/ion-channel regulatory domain at the C-terminus, connected by a hinge region. Mammalian CRISPs are involved in processes of reproduction, cancer, and immune responses. Snake venom CRISPs manifest some other activities, in particular, ion-channel inhibition. However, proteins of this family do not have a single target in a prey organism; moreover, no snake venom CRISPs have proven lethal to mammals. Their exact biological activity still remains unrevealed for many of them. At the same time, a key feature of this family of proteins is that, although they bind various target molecules, all affect cellular signaling [46].

Ablomin, triflin, and latisemin, CRISPs from the venoms of viperids and sea elapid, block high K^+^-induced contraction of arterial smooth muscle cells. Ablomin is supposed to inhibit the voltage-gated influx of extracellular calcium, which causes vascular contraction [47,48]. These proteins are believed to be L-type Ca^2+^-channel blockers.

On the contrary, natrin, a CRISP from *N. atra* cobra venom, induces a further contractile response in the endothelium-denuded thoracic aorta precontracted by a high K^+^ concentration. It has been shown to block high-conductance calcium-activated potassium (BKCa) channels that are involved in the regulation of the vascular tone [49].

### 2.8. Small Molecules and Nucleotidases

Purine adenosine, the most abundant non-proteinaceous component of many snake venoms, exacerbates venom-induced hypotension by activating adenosine A2 receptors in the vessel vasculature. Inosine, another purine present in snake venoms, can potentiate the vasodilatory effects of adenosine by acting both indirectly, activating A3 receptors on mast cells, and locally, as a prolonged agonist of several types of adenosine receptors [50]. Both catalytic (Asp49) and non-catalytic (Lys49) PLA_2_ myotoxins induce a rapid and massive release of ATP from affected myotubes [51]. Snake venom contains both phosphodiesterases and 5′-nucleotidases, which rapidly degrade ATP to adenosine [50,52].

We should note that there are a lot of other small molecules (e.g., taurine, *N*-acetylhistamine, oligopeptides, etc.) with an established (or proposed) hypotensive effect in snake venom (for a comprehensive work, see [50]). They are, however, either venom non-specific or low in content, or the locality of the effect is still questionable.

## 3. Toxins That Affect the Growth and Function of the Endothelium and the Regenerative Abilities of Blood Vessels

The formation of new blood vessels from preexisting ones, i.e., angiogenesis, is a sophisticated biological process that is strictly regulated by numerous factors and signaling pathways. Degradation of the BM surrounding existing blood vessels, primarily by matrix metalloproteinases, allows endothelial cells to proliferate and migrate toward angiogenic stimuli. First, these involve angiopoietins and other growth factors, the main one of which is VEGF. The latter interacts with specific receptors on endothelial cells and promotes their survival, differentiation and migration. A group of transmembrane proteins that are tightly involved in angiogenesis are the integrins, which play an important role in cell adhesion and in the communication between cells and the extracellular environment. To ensure the correct formation and remodeling of blood vessels, these factors act in a cooperative manner [53].

Snake venoms comprise toxins affecting all the stages of angiogenesis. Toxins that disorder the endothelial integrity (Section 4.2) should disrupt both vascular regeneration and neoangiogenesis, as discussed below.

The inhibition of angiogenesis in oncology may be considered as having a protective effect on the entire organism, while the induction of angiogenesis has a protective effect on an entire blood-supplied organ. Here, we would like to consider the local protective effect, i.e., protection of the walls of the blood vessel itself. It should be noted that in the literature, there are many examples of the cardioprotective effect of snake toxins, but, surprisingly, there are practically no examples of an angioprotective effect.

### 3.1. Vascular Endothelial Growth Factors

In the vasculature, receptors for VEGF (VEGFR-1 and -2) mediate angiogenic, mitogenic, and anti-apoptotic events. Mammalian VEGFs of types A, B, C, and D are able to induce angiogenesis. Snake venoms contain VEGFs of type F (VEGF-F). Despite the structural similarities to mammalian VEGFs, VEGF-F differs from them by the strength of its produced biological effects. For example, VEGF from *Trimeresurus flavoviridis* venom has 10-fold less mitotic activity than mammalian VEGF-A [54]. On the other hand, snake venom VEGF may be more angiogenic than mammalian VEGF. So, VEGF from *V. palestinae* venom demonstrates significantly higher activity in inducing human dermal microvascular endothelial cell (HDMEC) proliferation than human recombinant VEGF at the same concentrations [55].

Vammin, a VEGF from *V. ammodytes* venom, binds to VEGFR-2 more potently than mammalian VEGF-A; as well, it binds to the co-receptor neuropilin-1 but does not bind to VEGFR-1. VEGFR-2 is considered to be the main mediator of angiogenesis. Thus, vammin induces VEGFR-2/neuropilin-1 mediated proliferation [56]. Extensive studies have been carried out to clarify signaling pathways of vammin-induced angiogenesis. In HUVECs, it enhances the expression of genes associated with proliferation, migration, and angiogenesis more efficiently than VEGF-A. Vammin regulates a number of signaling pathways, including the expression of NR4A family nuclear receptors and regulators of calcium signaling and MAP kinase pathways. Ex vivo, in rabbit skeletal muscle, vammin induces prominent angiogenic responses, in which co-receptor binding is crucial for the switch in response from sprouting angiogenesis to vessel enlargement [56].

The increasing capillary permeability protein, ICPP, from *V. lebetina* manifests a potent capillary permeability activity (Section 4.3.3). In addition, when added to mouse embryonic stem cells or HUVECs, it is a powerful in vitro angiogenic factor. It has been demonstrated that in promoting in vitro angiogenesis of mouse embryonic stem cells, in activating p42/p44 MAPK, and in reinitiating DNA synthesis in HUVECs, ICPP is as effective as human VEGF_165_. Moreover, in establishing a vascular differentiated network, ICPP is even more active than VEGF_165_ [57]. It was shown that by binding to VEGFR-1, VEGF from *Protobothrops mucrosquamatus* venom promotes increased vascular permeability and neovascularization in the chicken chorioalantoic membrane, as well as proliferation and tissue factor production by endothelial cells [58].

An important phenomenon that induces cell motility is chemoattraction, and VEGF acts as a chemoattractant for endothelial cells during the progression of angiogenesis, stimulating their migration to the appropriate site of new, elongated capillaries [59]. Moreover, within 24 h in collagen type I gel, VEGF from *V. palestinae* efficiently stimulates radial migration of endothelial cells [59].

To overcome its immunogenicity as an exogenous protein, VEGF from *C. durissus terrificus* venom (CdtVEGF) has been PEGylated [60]. Both PEGylated and native CdtVEGF are able to induce migration and new vessel formation. An in vitro assay of tube formation on Matrigel demonstrated the angiogenic activity of CdtVEGF (20 ng/mL, 0.78 nM) through its ability to induce HUVECs to develop vessels. PEGylated CdtVEGF also stimulates HUVECs to form vessels, but this effect is not statistically significant; it also increases metabolic activity of HUVECs, which was demonstrated by the resazurin test [60].

Finally, it should be noted that VEGF, similarly to many other proteins in snake venom, can be represented by several active isoforms in venom from a single species. So, VEGF from *C. durissus collilineatus* venom is represented by six homologs, all of which are capable of inducing angiogenesis in HUVECs through tube formation on Matrigel. Moreover, two isoforms induce a more significant increase in tube formation, even when compared with basic fibroblast growth factor (bFGF) [61].

### 3.2. Disintegrins

**Figure 5:**
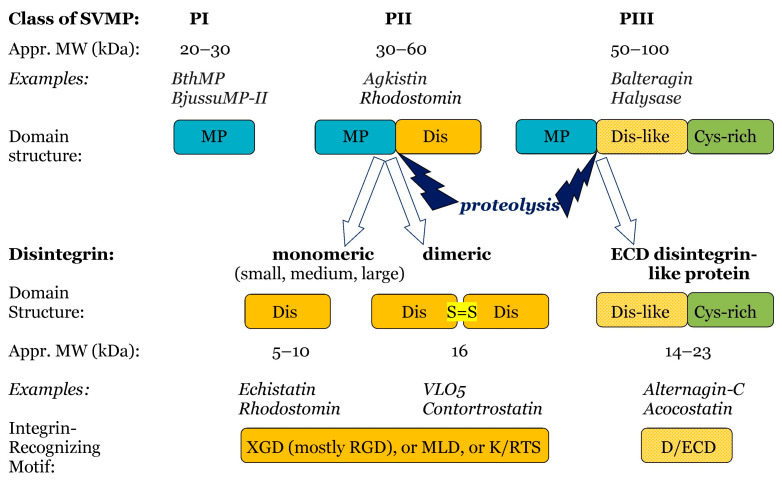
Relationship between SVMPs and disintegrins. MP, metalloproteinase; Dis, disintegrin; Dis-like, disintegrin-like; Cys-rich, cysteine-rich.

Snake venom disintegrins are derived from SVMPs (Figure 5). The disintegrins present in snake venoms are produced in two distinct ways:

(i) By proteolysis of class P-II SVMPs, with cleavage between the catalytic domain and the disintegrin domain. Such disintegrins are referred to as RGD (Arg-Gly-Asp)-dependent, with the sequence XGD (X-Gly-Asp), MLD (Met-Leu-Asp), or K/RTS (Lys/Arg-Thr-Ser) on the exposed surface of the loop that specifically binds to integrins on target cells [53]. The group of RGD-dependent disintegrins can exist as monomers—small ones with four disulfides, medium ones with six disulfides, and large ones with seven disulfides. Moreover, there are two subgroups of disintegrins that form homo- or heterodimers, with 10 disulfides within each subunit [53]. According to their disulfide bond patterns, medium and large disintegrins can be further subdivided into two and three sub-groups, respectively [62].
(ii) By proteolysis of class P-III SVMPs. This results in disintegrins composed of the ECD-containing disintegrin-like domain and cysteine-rich domains [53,62,63].

Disintegrins have been isolated mostly from Viperidae and Crotalidae snake venom, although venoms of certain species from the Atractaspididae, Elapidae, and Colubridae families also contain these polypeptides (see Table 1 in [62]).

The interaction of disintegrins with integrin receptors on endothelial cells is of pivotal importance in the modulation of angiogenesis. Thus, in a model of 10-day-old embryo chick chorioallantoic membrane (CAM), accutin, a disintegrin from *A. acutus* venom, demonstrates a potent antiangiogenic effect in vivo [64]. Salmosin, a disintegrin from the venom of the Korean snake *A. halys brevicaudus*, suppresses the bFGF-stimulated proliferation of bovine capillary endothelial (BCE) cells, affecting activation of integrin-mediated signaling molecules and producing changes in the cytoskeleton [65]. Obtustatin, a monomeric short KTS-disintegrin from the venom of *V. lebetina obtusa*, inhibits the microvascular endothelial cell proliferation induced by FBS and VEGF [55].

Snake venom disintegrins play a dual role in angiogenesis. They may act as antiangiogenic agents by inhibiting integrin binding and interfering with VEGFs; on the other hand, they may exert pro-angiogenic activities by enhancing integrin binding, stimulating cell migration and proliferation, and inducing neoangiogenesis [53]. A similar interference by disintegrin on the transmission of growth factor signaling should be underscored.

Most disintegrins, especially those affecting the RGD-dependent integrins αvβ3 and α5β1, as well as the α1β1 and α2β1 collagen receptors, have been shown to be potent inhibitors of angiogenesis (see table in [53]). The monomeric disintegrins kistrin and mambin bind only the αvβ3 integrin, whereas echistatin and flavoridin interact with both αvβ3 and α5β1 integrins on HUVECs [66]; lebestatin and obtustatin, KTS-disintegrins from *Macrovipera lebetina* venom, inhibit angiogenesis through the α1β1 integrin [63].

Some disintegrins can possess a more complicated mode of action. So, rhodostomin, a medium disintegrin from the venom of *Calloselasma rhodostoma*, inhibits angiogenesis by binding to integrins and inhibiting bFGF-induced proliferation of endothelial cells [67]. Saxatilin, from Korean snake *Gloydius saxatilis* venom, also significantly suppresses bFGF-induced HUVEC proliferation but has little effect on normal cell growth. Interaction of HUVECs with immobilized vitronectin is also inhibited by the binding of saxatilin to the αvβ3 integrin [68].

Recombinant disintegrin DisBa-01 from pit viper *B. alternatus* inhibits the adhesion to vitronectin in the human microvascular endothelial cell line-1 (HMEC-1) expressing the αvβ3 integrin and transiently inhibits their cell proliferation without direct cell toxicity [69]. In HUVEC 3D cultures, sprouting assays in collagen type I are decreased in normoxia upon DisBa-01 treatment, and VE-cadherin levels are diminished in HUVEC spheroids in hypoxia and upon DisBa-01 treatment [70]. In HMEC-1cells, DisBa-01 also suppresses VEGF release and decreases the expression of receptors of this growth factor [71].

A number of disintegrins inhibit angiogenesis. Their main targets have been shown to be integrins αvβ3 or α2β1 on HUVECs and integrins αvβ3 or α1β1 on HMECs [62]. In many cases of blocking angiogenesis in vivo, however, precise disintegrin target(s) have not been identified. On the other hand, certain disintegrins have been found to promote angiogenesis. For instance, jararhagin-C from *B. jararaca* venom, an ECD-disintegrin-like toxin composed of disintegrin and cysteine-rich domains, promotes angiogenesis by activating integrin receptors and stimulating endothelial cell migration, increasing the density of blood vessels and the synthesis of proangiogenic cytokines (VEGF and FGF) [72].

Another ECD-disintegrin-like toxin composed of these two domains, alternagin-C from *B. (Rhinocerophis) alternatus* snake venom, exhibits both pro- and antiangiogenic effects depending on its concentration. It inhibits VEGF signaling through VEGFR2 after binding to α2β1 integrin, resulting in impaired angiogenesis. Concentrations greater than 100 nM are considered antiangiogenic, whereas concentrations less than 50 nM are found to be proangiogenic both in vitro and in vivo [73,74]. The proposed explanation for this phenomenon involves the possibility of receptor recycling under the influence of low doses of integrin inhibitors. However, for molecules with higher molecular weight, such as disintegrins, one should also consider the release of metabolic (proteolytic) fragments that may have the opposite activity, stimulating proangiogenic cellular responses [63]. Alternagin-C as well as ALT-C PEP, a peptide derived from its sequence, induce angiogenesis in wounded rat skin. They induce the formation of new vessels and modulate the expression of growth factors, mainly VEGF and FGF1. So, stimulation of angiogenesis in an injured tissue by alternagin-C may be mediated by the expression of growth factors [75].

Alternagin-C stimulates in vitro HUVEC proliferation and suppresses the positive effect of VEGF or FGF-2 on the proliferation of these cells; this suggests a common mechanism for action for these proteins. The positive effect on HUVEC proliferation may be explained by upregulation of the VEGF gene and other growth factors in fibroblasts. Akt/PKB phosphorylation, a signaling event that takes part in angiogenesis and endothelial survival, is also highly activated by alternagin-C. So, alternagin-C functions as a survival factor, stimulating endothelial cell proliferation and adhesion [75,76].

Although the anti-platelet effect of disintegrins is beyond the scope of this paper, it should affect vasculature locally, at the point of thrombus formation. So, aggregating human platelets release serotonin, generate thromboxane A2, and dose-dependently induce vasoconstriction in de-endothelialized isolated rat thoracic aortas. Indeed, triflavin (1 µM), a monomeric RGD-disintegrin from *T. favoviridis* snake venom, significantly inhibits the platelet-induced vasoconstriction in de-endothelialized aortas. It markedly reduces the adhesion of platelets to the subendothelium in aortas, and at 2 µM, it significantly inhibits the release of serotonin and the formation of thromboxane A2 [77]. Thus, disintegrins, in particular triflavin, may be protective agents during angiospasms associated with thromboembolism. However, trigramin, another RGD-containing snake disintegrin, barely displays the above effects [77].

The interaction of the α9β1 integrin with NGF is also important in pathological angiogenesis. The α9β1 integrin antagonists, dimeric MLD-disintegrins VLO5 from *V. lebetina obtuse*, and bitisgabonin-2 from *Bitis gabonica* venom, block the function of human glioma microvascular endothelial cells in the presence of NGF but not physiological angiogenesis in the absence of NGF [78].

Moreover, the endothelium may not be the only target for snake disintegrins in the vascular system. Thus, both vascular endothelial cells and smooth muscle cells surrounding small blood vessels highly express the integrin α1β1. During normal vascular development, important processes include adhesion, migration, and proliferation of vascular smooth muscle cells. The adhesion of α1β1 integrin to collagen IV is selectively inhibited by recombinant jerdostatin, a short RTS-disintegrin from *P. jerdonii*, which dose-dependently suppresses α1β1-integrin-dependent HUVEC tube formation. Furthermore, the adhesion of rat aortic smooth muscle A7r5 cells to an immobilized CB3 fragment of collagen IV is also inhibited by jerdostatin in a dose-dependent manner, launching the rounding up, retraction, and, finally, detachment of these cells [79].

In summary, snake venom disintegrins evoke a broad spectrum of endothelial cell responses, but only few of them have been shown to exert direct apoptosis (Section 4.2.3).

### 3.3. Snake Venom Metalloproteinases

SVMPs are zinc-dependent metalloendopeptidases (EC 3.4.24). Together with so-called ADAMS (a disintegrin and metalloproteinase) enzymes, they are included in the adamalysins/reprolysins family of metalloproteinases within the Metzincin clan. SVMPs are characterized by a canonical sequence in the zinc-binding region at the catalytic site and by a conserved downstream turn, the Met-turn. Based on their domain composition, SVMPs are divided into three classes: PI, PII, and PIII. PI SVMPs are mature proteins contain only the metalloproteinase domain, whereas PII SVMPs, in addition to the catalytic domain, have a disintegrin domain. PIII SVMPs, in addition to the metalloproteinase and disintegrin-like domains, also contain a cysteine-rich domain [80,81]. Most PIII SVMPs include a disintegrin-like domain that has a conserved D/ECD motif, in contrast to the canonical RGD motif of the disintegrin domain in PII SVMPs [82]. Such differences in domain composition between different SVMP classes have significant effects on the mechanism of action of snake hemorrhagic toxins [81]. In turn, SVMPs may have some characteristic features within each class. They may be monomers or homo- or heterodimers, and they may undergo various types of post-translational modification. SVMPs not only generate other biologically active proteins that hydrolyze proteins in the extracellular matrix but they also have a very complex interrelationship with endogenous matrix metalloproteases. So, hydrolysis of type XV and XVIII collagen generates endostatin, an angiogenesis inhibitor, whereas the cleavage of the α-3 chain of type IV collagen by matrix metalloproteases releases tumustatin, another antiangiogenic molecule (for a review, see [83]).

P-I metalloproteinases from two *Bothrops* species arrest the cells in the G0/G1 phase and further induce both necrosis and apoptosis in endothelial cells, as judged by flow cytometry analysis. In vitro, P-I metalloproteinases exhibit significant antiangiogenic properties in 3D spheroid and Matrigel models by reducing sprout outgrowth and tube formation. Significant disruption of the peripheral vasculature in mice has been demonstrated using transplantation of agarose plugs containing metalloproteinase P-I [84].

BthMP from the venom of the snake *B. moojeni* is a P-I metalloproteinase that exhibits antiangiogenic properties in vitro and ex vivo via the VEGF pathway. At concentrations of 5 and 40 μg/mL, BthMP manifests no toxicity to HUVECs in the MTT assay and is unable to induce colony proliferation or necrosis. Interestingly, BthMP suppresses adhesion, migration and invasion of HUVECs in Matrigel. It also blocks angiogenesis in vitro by reducing the average number of nodules in cells treated with toxins and the number of tubules in a VEGF-dependent way. Moreover, in an ex vivo AR assay, BthMP suppresses the angiogenic process by reducing new vessel formation, decreasing the expression of the proangiogenic genes VEGF-A and ANGPT-1, as well as increasing the expression of the antiangiogenic gene SFLT-1. Overall, this toxin reduces the production of NO, a signaling molecule that promotes angiogenesis and VEGF modulation, and decreases by about 30% the VEGF-A protein expression in the supernatant of HUVEC cultures [85].

### 3.4. Snake C-Type Lectins

Lectins from snake venom are classified into true C-type lectins (CTLs) and C-type lectin-like proteins (CLLPs), also known as snaclecs. Both groups share the robust C-type lectin-domain fold. The molecular mass of CTLs is approximately 30 kDa, and they have a heterodimeric structure consisting of homologous α and β subunits linked by a disulfide bond. Unlike classical CTLs, with which they share sequence homology, snaclecs lack the Ca^2+^-binding capacity and carbohydrate-binding loop present in true CTLs and are therefore unable to bind sugars. Most snaclecs assemble in ordered supramolecular complexes with a high versatility of subunit numbers and geometric arrays [86,87].

Snake venom CTLs and snaclecs may affect the vascular endothelium. So, lebectin and lebecetin, two related 30 kDa heterodimeric CTLs from *M. lebetina* venom, act on integrins αvβ3, αvβ5, and α5β1. Lebectin acts as a very potent inhibitor (IC_50_ = 0.5 nM) of human brain microvascular endothelial cell (HBMEC) adhesion and migration on fibronectin by blocking the adhesive functions of both the α5β1 and αv integrins. In addition, lebectin strongly inhibits both HBMEC in vitro tubulogenesis on Matrigel (IC_50_ = 0.4 nM) and proliferation. Lebecetin reduces vascular sprouting by 85% at a concentration of 300 nM and totally inhibits sprouting and leads to the regression of pre-existing vascular sprouts at a concentration of 1.5 mM in mouse ARs in Matrigel. In vivo, lebectin displays potent antiangiogenic activity in both a chicken CAM assay and a Matrigel Plug assay in nude mice, and lebecetin efficiently reduces choroidal and retinal neovascularization by a single injection [88,89].

Vixapatin, a snaclec from *V. xantina palestinae* venom, is a selective α2β1 integrin inhibitor. It blocks proliferation of human dermal microvascular endothelial cells (HDMECs) at a concentration of 3 nM. At 1 µM, vixapatin decreases by 70% bFGF-induced physiological angiogenesis and reduces HDMEC tube formation by 75% in a Matrigel assay and C6 glioma-induced pathological angiogenesis by 94% in a shell-less embryonic quail chorioallantoic membrane assay [90]. ZK002, a 30 kDa heterodimeric snaclec from *Deinagkistrodon acutus* venom, shows antiangiogenic activity in multiple in vivo models. In HUVECs, ZK002 suppresses the activation of VEGF and VEGF-induced signaling and their related mediators, including HSP27, p38, eNOS, and LIMK. ZK002 also upregulates antiangiogenic TIMP3, the metalloproteinase inhibitor gene, and inhibits PPP3R2 and SH2D2A, components of the VEGF-induced signaling cascade [87].

BjcuL, a CTL from *B. jararacussu* venom, affects the behavior of endothelial cells. In a concentration- and carbohydrate-dependent manner, it interacts with HDMECs and reprograms the function of these cells, promoting a pro-inflammatory and pro-coagulant endothelial phenotype [91].

### 3.5. Kunitz-Type Serine Protease Inhibitor

Kunitz-type serine protease inhibitors are composed of typically 50–70 amino acids. A single chain adopts a conserved structural fold with two antiparallel β-sheets and one or two helical regions that are stabilized with three disulfide bridges presenting the protease-binding loop at the surface. Structurally, snake venom Kunitz-type inhibitors are related to other ones from animals, e.g., to the bovine pancreatic trypsin inhibitor. In addition to inhibiting serine proteases, they also perform other functions, such as modulating ion channels [92].

PIVL, a Kunitz-type serine protease inhibitor from *M. lebetina transmediterranea* venom, inhibits human vascular endothelial cell adhesion and migration onto fibrinogen and fibronectin in a dose-dependent manner without any cytotoxicity. PIVL increases microtubule dynamic instability in human microvascular endothelium cells (HMEC-1) and blocks angiogenesis in vivo and in vitro [93,94]. It impairs αvβ3 integrin by the RGD-like motif (41)RGN(43), which may be the reason for its antiangiogenic function.

### 3.6. Phospholipases A_2_

Some snake venom PLA_2_s are capable of inhibiting angiogenesis by blocking either integrins or the VEGF pathway in endothelial cells. Three acidic, nontoxic, Asp49 PLA_2_s from *Cerastes cerastes* and *M. lebetina* interfere with α5β1- and αv-containing integrins. Such an interaction results in impaired adhesion and migration of human brain microvascular endothelium cells (HBMECs) and HMEC-1 cells, as well as impaired HBMEC tubulogenesis on Matrigel in vitro. In an ex vivo CAM assay, these PLA_2_s strongly reduced vasculature development [95]. Some antiangiogenic properties of one of them, namely MVL-PLA2 from *M. lebetina* venom, are discussed below. It exhibits anti-*α*5*β*1 integrin activity and inhibits adhesion and migration of HMEC-1 cells in a dose-dependent manner while not being cytotoxic. Similarly to MVL-PLA2, its catalytically inactivated form significantly inhibits angiogenesis both in vitro and in vivo, suggesting that enzymatic activity is unimportant for this purpose. MVL-PLA2 treatment leads to the disturbance of the actin cytoskeleton and the allocation of *α*5*β*1 integrin, a critical regulator of angiogenesis and a major component of focal adhesions. MVL-PLA2 significantly increased microtubule dynamic instability in HMEC-1 cells by 40% [96].

Enzymatically active PLA2-I (Asp49) and inactive PLA2-II (Lys49) from the venom of *B. diporus* hindered tubulogenesis in tEnd cells. These cells are murine endothelial cell lines derived from a thymic hemangioma, which bears functional characteristics similar to those found in angiogenic endothelial cells. This inhibitory effect is more pronounced for the catalytically inactive toxin. Additionally, this PLA2-II, a PLA_2_-like myotoxin, effectively blocks fibronectin binding to the integrin receptor, offering a dual advantage over PLA2-I in interacting with the αVβ3 integrin [97].

The capability to bind to VEGFR-2 is a common characteristic of inactive PLA_2_ homologs from snake venom, known as myotoxins, but not of enzymatically active PLA_2_s. The C-terminal region of PLA_2_ is thought to interact with VEGFR-2 to inhibit angiogenesis and is responsible for disrupting membrane integrity. So, KDR-bp, an inactive PLA_2_ homolog from the venom of the eastern cottonmouth *A. piscivorus piscivorus*, interacts with the extracellular domain of VEGFR-2 (Kd of 10^−8^ M), and this results in the specific suppression of endothelial cell growth induced by VEGF_165_ [98]. BnSP-7, a Lys49 PLA_2_ analog from *B. pauloensis* snake venom, possesses the ability to inhibit HUVEC proliferation. It also inhibits the adhesion and migration of HUVECs and, in a VEGF-dependent manner, blocks angiogenesis in vitro. Finally, in an ex vivo AR assay, BnSP-7 is able to inhibit the sprouting angiogenic process [99]. BthTx-II, an Asp-49 PLA_2_ from *B. jararacussu* venom, suppresses cell proliferation, adhesion, and migration of HUVECs, as well as decreases VEGF levels in in vitro angiogenesis assays. In an ex vivo germination assay of the AR, BthTx-II showed the ability to inhibit the sprouting angiogenic process. Moreover, in HUVEC co-culture with triple-negative breast cancer cells, e.g., MDA-MB-231 cells, this toxin inhibited the migration and proliferation of HUVECs [100]. MjTX-II, a Lys49 PLA_2_-analog from *B. moojeni* venom, decreases VEGF expression and regulates the expression of pro- and antiangiogenic genes in HUVECs. Furthermore, in an AR model, MjTX-II inhibits ex vivo angiogenesis processes [101].

Without cytotoxic effects, crotoxin decreases the proliferation, adhesion, and migration of murine endothelial t.End.1 cells, thus blocking the angiogenesis. In a 3-D model in Matrigel, crotoxin also regulates endothelial cell projection and capillary-like structure formation. The primary mechanism of these effects is via the interference with cytoskeletal actin polymerization and the distribution of αv and α2 integrins. This mechanism may also include inhibition of the expression of FAK, a modulator of integrin binding to the extracellular matrix, and Rac1, a regulator of cell motility. Finally, in t.End.1 cells, crotoxin suppresses VEGF secretion and the expression of MMP-2 and MMP-9 associated with the degradation of the BM during angiogenesis [102].

Recently, an almost opposite protective effect of crotoxin on HUVECs has been reported [42]. So, it is not cytotoxic toward these cells at concentrations of up to 200 μg/mL and does not elicit apoptosis or necrosis. At the same time, crotoxin downregulates the LPS-induced production of some adhesion molecules, vasoactive factors, and interleukins in HUVECs, as well as protects the cells against H_2_O_2_-induced oxidative stress. Hence, in vitro, crotoxin displays an anti-inflammatory, antioxidative, and generally protective effect on HUVECs [42].

### 3.7. Cysteine-Rich Secretory Proteins

ES-CRISP from *Echis carinatus sochureki* venom interacts in a dose-dependent manner with various types of endothelial cells. In Matrigel, it inhibits endothelial cell proliferation, migration, and tube formation. ES-CRISP abrogates the neovascularization process induced by exogenous bFGF and VEGF in the embryonic quail CAM system. It can be internalized into the cytoplasm, where it regulates the expression of several factors characterized as regulators of angiogenesis at the mRNA level and blocks the activation of MAPK Erk1/2 induced by VEGF. By directly interacting with endothelial cells, ES-CRISP is a negative regulator of angiogenesis [103]. However, the target receptor for ES-CRISP has not yet been identified.

Natrin is a CRISP from cobra *N. atra* venom. To foster monocytic cell adhesion in a heparan sulfate- and Zn^2+^-dependent manner, it induces the expression of vascular endothelial cell adhesion molecules, such as E-selectin, vascular adhesion molecule-1, and intercellular adhesion molecule-1. Moreover, in HUVECs, it demonstrates the capability of activating the mitogen-activated protein kinases (MAPKs), including extracellular signal-regulated kinases (ERKs), c-Jun N-terminal kinase (JNK), and p38 MAPK [104]. Interestingly, while ES-CRISP is able to inhibit the proliferation of HUVECs and human glioma microvascular endothelial cells, it does not affect MAPKs, including the p38 kinase and stress-activated protein kinase (SAPK)/JNK. However, in HUVECs, it can inhibit the activation of ERK1/2 induced by VEGF.

### 3.8. L-Amino Acid Oxidases

Snake venom LAAO (EC 1.4.3.2) is a glycoprotein of 120–150 kDa in native dimeric form. It contains cofactor FAD, which determines the yellow color of crude venom, and Zn^2+^ ions, which are important for structural integrity. This enzyme stereospecifically deaminates an L-amino acid to an α-keto acid with the concomitant production of hydrogen peroxide and ammonia. LAAOs from various snake species display variable substrate amino acid specificities. In general, snake venom LAAOs are cytotoxic. Their interaction with the membrane increases the concentration of H_2_O_2_, which is important for the apoptotic and further necrotic activities of the enzyme.

Snake venom LAAO induces mainly hemorrhage and edema by assisting in the degradation of matrix proteins in endothelial cells and increasing the permeability of blood vessel walls, which results in fluid leakage from the capillaries into the interstitial space of the tissues [84]. LAAO causes cytotoxicity on endothelial cells by necrosis and apoptosis. The direct action of the protein or catabolic products on the plasma membrane in contributing to the degeneration of the cells results in necrosis, while reactive oxygen species (ROS) production leads to apoptosis [105].

By producing H_2_O_2_ in endothelial cells, venom LAAOs induce caspase-mediated apoptosis driven by ROS, which activate caspase-3 and caspase-9. Apoxin I, an LAAO from *C. atrox*, induces apoptosis in HUVECs and rat endothelial NK-3 cells by giving rise to the condensation and fragmentation of DNA [106]. In HUVECs, BF-LAAO, an LAAO from the venom of *B. fasciatus*, inhibits cell growth and promotes apoptosis in a dose-dependent manner [107]. In human endothelial cells, LAAO-II from *B. jararacassu* venom significantly reduces viability and survival and induces DNA damage by increasing ROS levels [108].

LAAOs from two *Bothrops* species arrest the cells in the G0/G1 phase approximately 20% more actively than metalloproteinases P-I from the same venoms and further induce both necrosis and late apoptotic modes of cell death in HUVECs, as judged by flow cytometry. In vitro, LAAOs exhibit significant antiangiogenic properties in 3D spheroid and Matrigel models by reducing sprout outgrowth and tube formation by HUVECs and the rearrangement of HUVECs on Matrigel within 6 h upon treating with bFGF. LAAO-induced cytotoxic effects are more prominent in endothelial cells than fibroblasts [84].

### 3.9. Inhibition of Neoangiogenesis as a Contributor to the Anti-Tumor Effect

The anti-tumor effect, as such, has been described for a variety of snake venom toxins; its mechanism with respect to tumor cells can be quite different and may involve induction of cell lysis (cytotoxicity), apoptosis, anti-migration, anti-adhesion, and so on (for a review, see [109]). The anti-tumor effect may also be related to the impact of snake toxins on blood vessels. As a rule, it is associated with the antiangiogenic effect of toxins on pathological neoangiogenesis during tumor growth. Angiogenesis is a prerequisite in solid tumors for enabling their growth and metastasis. A single vessel can support about 100 tumor cells. The overexpression of angiogenic factors and the downregulation of angiogenic inhibitors, known as the “angiogenic switch”, is particularly essential for tumor progression [102]. If there is no adequate blood supply, tumor growth is retarded. Therefore, toxins impeding angiogenesis as discussed above (for example, by inducing endothelial apoptosis and so on) may decelerate solid tumor development. In particular, inhibition of angiogenesis has been found to be the principal or significant cause for the decrease in tumor growth when using snake toxins from the following groups: disintegrins, SVMPs, PLA_2_s, LAAOs, and CLLPs (summarized in a review [109]).

It should be emphasized that the antiangiogenic effect is not always the key reason for the anti-tumor effect of a snake toxin. For example, crotoxin is not only an antiangiogenic but also a well-known anti-tumor agent. On the one hand, its anti-tumoral effect is due to its direct impact on tumor cells; it activates apoptosis, induces cell cycle arrest, inhibits metastasis, and decreases tumor growth in different tumor types. On the other hand, crotoxin also affects the tumor microenvironment; it modulates tumor-associated fibroblasts, immune cells, and, finally, endothelial cells [110]. That is, inhibition of angiogenesis is only one of several mechanisms of crotoxin anti-tumoral action.

Cathelicidin BF-30, an antibacterial 30-amino acid polypeptide from *B. fasciatus* krait venom, reduces melanoma B16F10 cell growth and simultaneously reduces the vascular network around the tumor. The latter is due to BF-30 dose-dependent suppression of transcription of the VEGF gene and protein expression in B16F10 cells [111]. However, the whole set of data presented by the authors [111] indicates an overall cytotoxic effect of BF-30 on melanoma cells, and the inhibition of VEGF-upregulated angiogenesis is, rather, a consequence of this cytotoxicity but not its reason.

### 3.10. Antiangiogenic Effect of α-Bungarotoxin

Although several subtypes of nicotinic acetyl choline receptor (nAChR) are expressed on endothelial cells, the α7 homopentamer is the predominant nAChR involved in angiogenesis. TFT α-bungarotoxin, a relatively selective α7-nAChR antagonist from krait venom, completely and reversibly inhibits nicotine-evoked endothelial network formation by HUVECs and HMVECs in Matrigel [112]. A study of the influence of nicotine on human embryonic stem-cell-derived endothelial cells have shown that nAChR activation upon hypoxia promotes the upregulation of VEGF and bFGF gene expression, and α-bungarotoxin abrogates this effect [113]. Such an action of α-bungarotoxin may be helpful in cases when angiogenesis is undesirable—for example, in neovascular age-related macular degeneration. Thus, in vivo, it reduces the choroidal neovascularization in this disease, both with and without the addition of nicotine [114].

## 4. Toxins That Damage Vascular Wall Structures

### 4.1. Toxins That Damage the Lipid Bilayer of the Cell Membrane (Direct Cytotoxicity)

Damage to the lipid bilayer of the cytoplasmic membrane would obviously lead to dramatic consequences for the cell, resulting in its death (cytotoxicity). The following classes of snake venom toxins can directly damage the lipid bilayer: cytotoxins, PLA_2_s (including enzymatically inactive myotoxic PLA_2_s), and short myotoxins. Most of the work studying such damage has been performed on tumor cell lines for obvious utilitarian reasons, with vascular epithelial cells used only occasionally for toxicity comparisons. Cardiomyocytes and muscle myocytes are commonly used to assess the myotoxicity of snake toxins; we have been unable to find any examples of studies of lipid bilayer disruption in myocytes of the muscle wall of blood vessels. However, it can be assumed that, in general, compounds with cytotoxic activity have low or no cellular selectivity. Therefore, the general mechanisms of action studied in tumor cells, myocytes, and cardiomyocytes may be extrapolated with some caution to vascular wall cells.

#### 4.1.1. Cytotoxins

Snake venom CTXs are hydrophobic, positively charged polypeptides, which suggests that they target the lipid bilayer of the cell membrane. The name “cytotoxins” itself indicates their ability to kill various types of cells, including muscle and endothelial cells.

Despite snake venom CTXs preferring membranes of cardiomyocytes and dividing cells, they exhibit rather non-specific cytotoxicity. To induce cytotoxicity, they promote cell cycle arrest and activate cell death pathways, including both necrotic and apoptotic ones, as well as necroptosis (a form of programmed necrosis) in both cancerous and non-tumor cell lines [115]. The mechanism of cell death includes CTX binding to the phospholipid bilayer (with a preference for acid phospholipid content), internalization, lysosomal damage, and interaction with cardiolipin of the outer sheet of the mitochondrial membrane, with a violation of energy metabolism [116]. It should be said here that CTXs cause cell death in significantly higher concentrations, and afterward, they cause a strong functional response in the vasculature (discussed in Section 2.4.1).

The cytotoxicity of Chinese cobra venom CTX (cardiotoxin) on the endothelium was investigated in cultured rabbit aortic endothelial cells. In Hank’s buffered saline solution with Ca^2+^, cardiotoxin (1–30 µM) caused cell necrosis and cell death within 20 min in a concentration-dependent manner, as determined by a trypan blue exclusion test [25].

#### 4.1.2. Phospholipases A_2_

PLA_2_ catalyzes the cleavage of the ester bond of phospholipids. As biological membranes are composed of phospholipids, it is believed that PLA_2_s alter the membrane’s fluidity and cause membrane permeabilization, which ultimately leads to cell death. In general, acidic PLA_2_s possess a higher IC_50_ than basic PLA_2_s. The acidic PLA_2_s are less cytotoxic than catalytically inactive K49 basic PLA_2_s. The basic PLA_2_ homologs, K49 and S49 PLA_2_s, are responsible for many Ca^2+^-independent biological activities [117].

The mechanism of PLA_2_ cytotoxicity has been studied using various tumor cell lines. The unified mechanism and exact pathways of implementing PLA_2_ cytotoxicity are still a matter of debate, but the overall picture is as follows: The C-terminal region of PLA_2_ is thought to be responsible for disrupting membrane integrity; it can also bind to vascular endothelial growth factor receptor-2 (VEGFR-2) to inhibit angiogenesis. PLA_2_-induced phospholipid metabolism may lead to the release of reactive oxygen species. Elevated oxidative stress results in the activation of cell death pathways. It might involve the downregulation of anti-apoptotic proteins such as Bcl2, Bcl-XL, and c-FLIP, an increase in pro-apoptotic BAD expression, and the activation of caspase 3 to disturb the cell cycle and cause apoptosis. PLA_2_ also can induce genotoxic effects and DNA damage (reference [117] and refs. therein).

How correct is it to transfer the data obtained from tumor cells to normal vascular wall cells? The results of studying the cytotoxicity of PLA_2_ against tumor cells and HUVECs under the same conditions show that it is possible, but some caution is needed.

Enzymatically inactive Ser49 PLA_2_ analogs purified from the four *Echis* species have LC_50_ values against A549 cells in the range of 2.9–8.5 μM and LC_50_ values against HUVECs that are almost in the same range (2.5–12.2 μM). This non-significant difference indicates that the Ser49 PLA_2_ analogs manifest no differential anti-tumor activity [118].

At the same time, Cc-PLA2-II, a group-II-secreted PLA_2_ from Saudi *C. cerastes gasperetti* snake venom, exerts a significant dose-dependent cytotoxic anti-tumor effect against six human cancer cell lines without the cytotoxic effect seen in normal HUVECs [119]. Crotoxin, another PLA_2_ from group IIA that is known to be cytotoxic to various tumor cells, is also not cytotoxic to HUVECs [42]. These are examples of PLA_2_s with manifested anti-tumor toxicity without direct damage to endothelial cells.

In contrast, Bothropstoxin-I, a myotoxic Lys49 PLA_2_ from *B. jararacussu* snake venom, appears to be more toxic to normal cells. So, it induces a decrease in HUVEC cell viability in vitro at concentrations of 1 μg/mL and above, whereas in DU-145 cancer cells, it does so at concentrations of 10, 25, and 50 μg/mL. The comet assay shows that all BthTX-I concentrations promote an increase in DNA damage in both HUVEC and DU-145 cells [120].

Based on the single examples given, one should not hastily conclude that preferential toxicity toward tumor cells or vascular epithelial cells depends on whether the toxin belongs to Ser49-, or Asp49-, or Lys49-PLA_2_. It is obvious that the single examples available to date are not enough to draw such a firm conclusion.

### 4.2. Toxins Affecting the Cell Interaction with the Extracellular Matrix (ECM)

The adhesion of cells to the matrix is largely due to the interaction of integrins and cell surface proteins with their counterparts: proteins of the extracellular matrix and neighboring cells. Some snake venom toxins bearing the sequence XGD/MLD or K/RTS are capable of impeding these interactions by binding to integrins. Such venom components, mainly belong to SVMPs or to disintegrins released by the proteolysis of PII and PIII SVMPs. In addition, several snake venom PLA_2_s and snaclecs also display similar properties, as well as certain members of other toxin groups. Most published studies involve the inhibition of platelet aggregation and/or tumor growth (see reviews [109,121]); these will not be considered in this paper. At the same time, there are many examples of snake toxins blocking the integrins in the endothelium of blood vessels. The blocking of some types of integrins leads to impaired adhesion of epithelial cells, their detachment, loss of functional shape (rounding), and apoptosis, leading to damage to the vascular wall. Endothelial cells express a subset of mammalian integrins, which include the fibronectin receptors α4β1 and α5β1, the collagen receptors α1β1 and α2β1, the laminin receptors α3β1, α6β1, and α6β4, the osteopontin receptor α9β1, as well as the vitronectin receptors αvβ3 and αvβ5 [122].

#### 4.2.1. Disintegrins

As discussed above (Section 3.2), snake disintegrins may impede vascular endothelial integrin binding to components of the extracellular matrix (ECM), such as fibrinogen, fibronectin, and vitronectin, with various degrees of selectivity and affinity. The adhesion of HUVECs to vitronectin is significantly inhibited by monomeric disintegrins—echistatin, kistrin, flavoridin, and mambin—but not bitistatin. Echistatin and flavoridin have a modest inhibitory effect on HUVEC adhesion to fibronectin. In solid-phase assays, echistatin, kistrin, and flavoridin interact with high affinity with immobilized αVβ3 integrin. Adhesion of HUVECs to vitronectin and fibronectin leads to cell spreading, whereas cells adhering to immobilized kistrin show abnormal morphology, and cells adhering to echistatin remain globular [66]. The disintegrin accutin from *A. acutus* venom specifically inhibits the binding to HUVECs by a monoclonal antibody recognizing integrin αVβ3. In functional studies, accutin demonstrates inhibitory effects on HUVEC adhesion to immobilized fibrinogen, fibronectin, and vitronectin [64].

On the surface of bovine capillary endothelial cells, salmosin, a disintegrin from the venom of the Korean snake *A. halys brevicaudus*, competes with the ECM for direct binding to integrin αVβ3. In this way, it disassembles cortical actins at focal adhesions and induces the rounding and detachment of cells. In bovine capillary endothelial cells, salmosin inhibits activation of focal adhesion kinase (FAK) and reduces the expression of paxillin and p130CAS [65].

Obtustatin, a monomeric short KTS-disintegrin from *V. lebetina obtusa* venom, was applied onto HDMECs as a specific inhibitor of α1β1 integrin. HDMECs treated with a high concentration of obtustatin (2 μM) significantly change their shape and begin to detach, showing typical apoptotic morphology [55].

#### 4.2.2. Snake Venom Metalloproteinases

SVMPs impair the connective tissue components responsible for blood vessel structural integrity. SVMPs cleave various types of ECM proteins, including laminin, nidogen, enactin, type IV collagen, fibronectin, proteoglycans, and others, as well as degrade structurally important BM components, such as perlecan and, especially, type IV collagen, and possibly type VI and type XV collagen. This protein degradation is followed by the mechanical disruption of vessels due to hemodynamic biophysical forces, which is a so-called “two-step” process (see reviews [81,123,124]). Such a disruption may result in elevated vascular permeability and hemorrhage (discussed in Section 4.3). Moreover, SVMPs may activate some other endogenous proteases, in particular, caspases-3 and -7, cathepsins D and E, granzyme B, and matrix MPs 2 and 9, which may lead to further adverse consequences [125]. Balteragin, a PIII SVMP from the venom of *B. alternatus,* detaches tEnd endothelial cells and induces a gradual rounding of the cells, which eventually detach from the monolayer, with some evidence of shrinkage, chromatin condensation, and membrane blebbing [126].

Some SVMPs, like mammalian ADAMs, may cleave the TNF-α precursor. TNF-α upregulates the expression of adhesion molecules on endothelial cell surfaces. BjussuMP-II, a P-I SVMP from *B. jararacussu* snake venom, interferes with HUVEC adhesion and promotes their detachment but is not toxic to these cells at the range of concentrations or durations of treatment used. Phorbol 12-myristate 13-acetate stimulates TNF-α release by HUVECs; however, this effect is not found for BjussuMP-II, which cleaves TNF-α [61].

#### 4.2.3. Vascular Apoptosis-Inducing Proteins and Disintegrins Evoke Endothelial Cell Apoptosis

A group of SVMPs from Viperidae snake venoms is peculiar due to their ability to induce death of vascular cells by apoptosis, so, they are named Vascular Apoptosis-inducing Proteins (VAPs, as the first member of the group was named). The first described VAP did not trigger the detachment of cells and did not induce necrotic cell death. But it evoked extensive death of HUVECs by an apoptotic pathway, which was manifested by the formation of blebs on the cell surface and cell shrinkage. All this resulted in the generation of apoptotic bodies and was accomplished by DNA fragmentation [127]. All the VAPs described later have similar effects. In general, they digest proteins of the ECM (see SVMPs in Section 4.2.2) that are crucial for cellular viability, and this triggers apoptosis. They may provoke detachment similarly to the effect of VAP graminelysin I on HUVECs [128] but do not appear to kill cells directly. Experiments with antibodies have shown that homodimeric VAP (VAP1) from *C. atrox* venom induces apoptosis in HUVECs through the α3, α6, and β1 integrins and a transmembrane protein, CD9, that is usually associated with integrin receptors [129].

These SVMPs may be of the monomeric P-I type, like the hemorrhagic rubelysin from *C. ruber ruber* rattlesnake venom and okinalysin from *Ovophis okinavensis* himehabu venom [130,131] or the non-hemorrhagic rubelase from *C. r. ruber* [130] and graminelysin from *T gramineus* pit viper venom [128]). They may belong to the P-III type, like monomeric halysase from *G. halys* venom [132], homodimeric VAP (VAP1) [127], and heterodimeric VAP2 from *C. atrox* venom [133]). Hemorrhagic VAPs produce more pronounced apoptotic effects on both endothelial (HUVECs and human pulmonary artery endothelial cells) and aortic smooth muscle cells than non-hemorrhagic VAPs [131].

Disruption of the interaction between integrins and integrin receptors can apparently lead to impaired cell attachment with subsequent cell death. Expectedly, some disintegrins without metalloprotease activity can evoke apoptosis. As discussed in Section 4.2.1, some disintegrins detach cells. Further, accutin potently induces HUVEC apoptotic DNA fragmentation, as examined by electrophoretic and flow cytometric assays [64]. The mechanism of obtustatin action is related to the blocking of microvascular endothelial cell proliferation, which undergoes caspase 8/3-dependent apoptosis [55]. BCE cells treated with salmosin eventually undergo caspase 3-dependent apoptosis [65].

Apoptosis is also described as the main effect for some disintegrin-like proteins. So, recombinant acocostatin, a truncated P-III SVMP from *A. contortrix contortrix* composed of disintegrin-like and cysteine-rich domains, induces apoptosis in HUVECs, as determined by Annexin V-FITC and chromatin fragmentation assays. The authors [134] consider αvβ5 receptors as the main target for acocostatin.

Along with ECM proteins, integrins α1β1 and α5β1 are also specifically hydrolyzed by halysase, a P-III SVMP. Caspase-3 activation and Bcl-X(L)/Bax downregulation underlie halysase-induced HUVEC apoptosis. Apohalysase, which has no metalloprotease activity, is also capable of inducing apoptosis. Thus, the disintegrin-like domain and the protease domain of halysase cooperatively contribute to the induction of apoptosis in endothelial cells [132].

#### 4.2.4. Phospholipases A_2_

Mammalian PLA_2_s are identified as ligands for the αvβ3 and α4β1 integrins, and their receptor-binding sites have already been localized [135]. Some snake venom PLA_2_s are implied to bind to and interfere with αv (αvβ3), α2, and α5β1 integrins, which result in loss of adhesion of vascular endothelial cells [95,97,102]. As an examples, C-PLA2-1 and CC-PLA2-2 from *C. cerastes* venom display anti-integrin activity. They efficiently inhibit HBMEC adhesion and migration to fibrinogen and fibronectin in a dose-dependent manner. This anti-adhesive effect is mediated by α5β1 and αv-containing integrins [136].

Crotoxin, a PLA_2_ of group IIA, inhibits capillary-like tube formation on 3D Matrigel and impairs murine endothelial t.End.1 cell adhesion to different ECM components as well as their proliferation and migration. However, these effects do not result in loss of cell viability. Cytoskeletal actin polymerization (F-actin) and the reduction of αv and α2 integrin distribution are related to the inhibition of cell adhesion to type I collagen and, to a lesser extent, fibronectin and laminin. Inhibition of the actin-related protein 2/3 (Arp 2/3) complex as well as focal adhesion kinase (FAK) and Rac1 (GTPase) signaling proteins accompany these processes [102].

An interesting synergistic effect on the detachment of tEnd endothelial cells was reported for SVMP and PLA_2_ from the same *Bothrops* venom [126]. An acidic PLA_2_ (Ba SpII RP4) lacks its own toxicity and detaching activity toward endothelial cells. At the same time, the detaching effect of the SVMP is enhanced significantly by PLA_2_, even at a concentration as low as 1 μg/mL. This synergistic effect is not affected by the inhibition of the PLA_2_ enzymatic activity by p-bromophenacyl bromide, suggesting that the observed enhancement does not depend on phospholipolytic activity and, instead, an interaction of PLA_2_ with endothelial cell plasma membrane may be responsible for this effect.

Along with PLA_2_s, some snaclecs, in particular, those from *Lebetina* viper venom, as well as certain other toxins, may demonstrate a disintegrin-like activity as well, and if so, they should affect intercellular interactions. However, there are no data about their destructive activity against vascular wall structures. Therefore, they are considered in Section 3.4 and Section 3.5.

### 4.3. Toxins That Increase Vascular Permeability

Degradation of the ECM by SVMPs and hyaluronidases promotes the diffusion of venom components and can contribute to cellular damage indirectly by affecting the stability of endothelial cells in capillaries. Vascular permeability may result in hemorrhage and edema. Edema, however, may evolve not only by increased vascular permeability but also for other reasons under many pathological conditions. So, here, we discuss data only on toxin-caused vascular permeability, which may or may not be accompanied by edema. It should be noted that snake venom kininogenases may increase capillary permeability indirectly by liberating bradykinin.

#### 4.3.1. Snake Venom Metalloproteinases

SVMPs play central roles in hemorrhage caused by snake venoms. One of the first reports on detailed ultrastructural alterations in muscle tissue morphology injected with hemorrhagic SVMPs and crude venom from *C. atrox* appeared in 1978 [137]. In the affected capillaries, endothelial cells exhibited a drop in the number of pinocytotic vesicles, intracellular swelling, dilatation of endoplasmic reticulum, formation of blebs, and, what is most crucial, disruption and formation of gaps between the cells through which erythrocytes ran away. While the basal lamina was impaired to some extent, the intercellular junctions between endothelial cells were not influenced. This mechanism was named “hemorrhage per rhexis” [137].

In contrast, “hemorrhage per diapedesis” may occur if the affected microvessels are venules, in which erythrocytes escape through widened intercellular junctions. Both extravasation mechanisms, i.e., per rhexis and per diapedesis, are likely to occur in tissues as a result of the action of hemorrhagic SVMPs [81,124]. Ultrastructural studies of skeletal muscle provide strong evidence that the per rhexis mechanism predominates, and that the primary site of action of SVMP is the capillary network rather than the venular part of the microvasculature.

In vivo, acute damage to endothelial cells by SVMPs occurs within a few minutes. In contrast, in cell culture, there is no such cell damage over several hours [138]. Instead, the main effect is detachment and rounding of the cells. To explain SVMP-induced hemorrhage in vivo, a “two-step” mechanism was suggested [123]. Primarily, SVMPs destroy the BM and degrade adhesion proteins, thereby weakening the capillary wall and disrupting the interaction between endothelial cells and the basal layer. Then, transmural pressure acting on the weakened capillary wall produces distention. As a result, the endothelial cells become extremely thin, to the point that the integrity of the capillary wall is compromised in some areas where extravasation occurs. Moreover, endothelial cells become more susceptible to blood-flow-dependent shear stress, further contributing to capillary wall degradation [123].

P-III SVMPs, which contain metalloproteinase, disintegrin-like and cysteine-rich domains, demonstrate more potent hemorrhagic activity than P-I SVMPs, which have only the metalloproteinase domain. SVMPs degrade various components of the BM and are also capable of hydrolyzing proteins of the endothelial cell membrane, such as integrins and cadherins taking part in cell–matrix and cell–cell adhesion. In addition, SVMPs bind to endothelial cell integrins via their disintegrin-like and cysteine-rich domains, preventing their adhesion to the extracellular matrix [123]. The binding of P-III SVMPs to collagens and the α2β1 integrin is mediated by their disintegrin-like and Cys-rich domains, respectively. Furthermore, the cysteine-rich domain controls SVMP binding to type I collagen and several proteins containing von Willebrand factor A domains [124].

Thus, P-I SVMPs are implied to be less hemorrhagic compared with P-III ones. However, P-I SVMPs can have a very destructive effect on the vascular wall. BaP1, a P-I SVMP from *B. asper* venom, and CsH1, a P-III SVMP from *C. simus* venom, were compared in experiments on muscle microvasculature in ex vivo and in vivo models [139]. CsH1 indeed exhibits a stronger effect on type IV collagen than BaP1, while BaP1 disrupts the endothelial barrier in post-capillary venules and increases vascular permeability. Moreover, BaP1 increases the size of the gaps between pericytes in post-capillary venules and creates new gaps between smooth muscle cells in arterioles under ex vivo conditions. These effects are not observed for CsH1. Moreover, while the action of CsH1 is more directed to the BM of microvessels, the effects of BaP1 are widely spread to other microvascular components [139].

Hemorrhagic factor 3 (HF3), a P-III SVMP from *B. jararaca*, induces severe local hemorrhage at picomolar doses in a murine model. Different proteoglycans of endothelial glycocalyx and the platelet-derived growth factor receptor are digested by SVMPs, which results in the disruption of microvasculature integrity and the generation of hemorrhage [140].

Both non-hemorrhagic and hemorrhagic SVMPs hydrolyze some proteins in the BM and associated ECM. Nevertheless, only hemorrhagic SVMPs are capable of destroying microvessels; the mechanisms underlying this functional difference remain largely unexplored. So, both the non-hemorrhagic leucurolysin-a (leuc-a), P-I SVMP from the venom of *B. leucurus*, and the hemorrhagic BaP1, P-I SVMP from the venom of *B. asper*, are capable of cleaving BM proteins. Both enzymes hydrolyze perlecan, nidogen, and laminin, albeit BaP1 does so at a higher rate, especially for type IV collagen. The variable ability of these SVMPs to degrade key BM and associated ECM substrates in vivo may explain the differences in their hemorrhagic activity. This particularly concerns perlecan and several non-fibrillar collagens, which play a mechanical stabilizing role in microvessel structure [141].

#### 4.3.2. Disintegrins

As disintegrins may detach endothelial cells and thus destroy the vessel wall, they might be anticipated to promote vascular permeability. Surprisingly, there is no such evidence for snake disintegrins, although there are a lot of data about their effects on the detachment and apoptosis of endothelial cells. We were unable to find any papers describing an increase in vascular permeability under the influence of disintegrins

At the same time, there is an example of the opposite action of integrin. Fc-saxatilin, a fusion protein of an immunoglobulin Fc domain with saxatilin from *G. saxatilis* venom, specifically binds to integrin αvβ3 in endothelial cells and can prevent vascular leakage under hypoxic or ischemic conditions. In mouse brain microvascular endothelial cells, hypoxia leads to an increase in cell permeability to FITC-dextran and an enhancement in MMP-9 activity as well as to the downregulation of occludin, a tight-junction protein, and to the activation of FAK, a downstream signaling molecule in integrin-dependent signal transduction. Fc-saxatilin weakens all these effects. In mice, Fc-saxatilin also prevents the vascular leakage of Evans Blue in the ischemic brain induced by occlusion of the middle cerebral artery [142]. Moreover, Fc-saxatilin blocks VEGF-induced permeability in HBMECs under ischemia. It also inhibits the activation of Src and FAK, downstream signaling proteins of VEGF in the induction of endothelial permeability (the next section), and recovers the downregulation of claudin-5, a tight-junction protein [143].

#### 4.3.3. Vascular Endothelial Growth Factors

The vascular permeability increase induced by type-F VEGFs from snake venoms through specific receptors is rapid, transient, and reversible [31]. VEGFs of types A, B, C, and D regulate the formation and permeability of blood vessels in vertebrates. Snake VEGFs of type F are structurally and functionally similar to vertebrate ones, especially to VEGF-A, and they are considered important contributors to the envenoming due to their capacity for increasing vascular permeability. VEGFs that have been isolated from snake venom and functionally characterized have been discussed in a recent review [31]. These have all been isolated from viperid venoms and shown to increase vascular permeability, and only some of them display other activities typical for the VEGF family. Although VEGF-Fs from different venoms are similar in their effects on vascular permeability, they may differ in their affinity for their specific receptors and in the expression of other types of activity.

Thus, the first VEGF-F purified from *V. aspis aspis* venom was initially discovered by monitoring the hypotensive effect associated with its binding to heparin. This protein immediately turned out to be more active in increasing capillary permeability than even VEGF-A [33]. TjsvVEGF from *T. jerdonii* venom demonstrates low affinity to heparin [144], and VEGF from *G. tsushimaensis* venom (GtVF) displays no affinity to heparin [145]. At the same time, both these proteins induce strong capillary permeability.

Most of the characterized VEGF-Fs preferably bind VEGFR-2, while only a few bind VEGFR-1 [54,58]. The latter have, so far, been found in pit viper venoms and include TfsvVEGF from *T. flavoviridis* and Pm-VEGF from *T. mucrosquamatus*. Thus, TfsvVEGF has nearly 10-fold less mitotic activity than VEGF_165_, the predominant isoform of human VEGF-A, but has a similar effect on vascular permeability. TfsvVEGF binds VEGFR-2 weakly and induces its autophosphorylation almost 10-fold less effectively than VEGF_165_; however, it binds VEGFR-1 and induces its autophosphorylation to almost the same extent as VEGF_165_. This unique ability to bind to VEGFR-1 and VEGFR-2 results in the dominant activity of TfsvVEGF being the induction of vascular permeability without angiogenesis, followed by regeneration of damaged tissue [54].

IC1 (increasing capillary 1) and IC2 (increasing capillary 2), isoforms of svVEGFs from the *M. lebetina* venom, are able to promote capillary permeability through the binding of VEGFR-2 in the nanomolar range, although they are also able to bind to neuropilins NPR-1 and NPR-2, co-receptors for growth factors expressed in the vascular systems, in the micromolar range [146].

The phosphorylation of VEGFR appears to be crucial for the effects of VEGF-F. So, all biological actions, including capillary permeability in mice, of increasing capillary permeability protein (ICPP), which is a VEGF from *V. lebetina* venom, are fully inhibited by 1 µM ZM317450, a specific VEGF receptor tyrosine kinase inhibitor [57].

#### 4.3.4. Hyaluronidase

Hyaluronidase digests the glycosaminoglycan hyaluronan in the ECM to generate potentially immunopathological tetra- and hexasaccharides. It is considered the main snake venom spreading factor. To deliver toxins, it increases the permeability of tissues and blood capillaries. Endothelial dysfunction induced by these enzymes may occur, for example, via lipid mediators elicited by low-molecular-weight products of hyaluronan degradation [147].

#### 4.3.5. Crotaline Cysteine-Rich Secretory Proteins

Hellerin from *C. oreganus helleri* is the first CRISP with established effects on blood vascular permeability and the permeability of monolayers of blood and lymphatic endothelial cells. Hellerin induces morphological alterations in HUVECs, such as rounded cell shape and detachment. Presumably, these effects may be related to a 20% decrease in F-actin levels under the influence of hellerin. Trans-capillary leakage occurs 30 min after mice have been subcutaneously injected with hellerin, but the vascular permeability is approximately half of that produced by VEGF-A [46,148].

Following the hellerin discovery, five CRISPs have been isolated from venoms of *C. adamanteus, C. atrox, C. horridus*, *C. s. scutulatus*, and *A. p. piscivorus* from the Crotalinae family. Of these, Css-CRISP from *C. s. scutulatus* and App-CRISP from *A. p. piscivorus* produce the most prominent increase in vascular and endothelial permeability in human dermal lymphatic (HDLECs) and blood endothelial cells (HDBECs) while manifesting no cytotoxicity and without detectable morphological or cell viability changes. In HDBECs, both Css-CRISP and App-CRISP produce significant phosphorylation of Src and mTOR, respectively, and induce caveolin-1 expression. In HDLECs, App-CRISP significantly enhances JNK and Akt phosphorylation, while Css-CRISP significantly downregulates the expression of phospholipase C-γ and N-cadherin. These results suggest that crotaline CRISPs increase the endothelial permeability in HDLECs and HDBECs through different pathways [149].

#### 4.3.6. γ-Bungarotoxin

This toxin belongs to the non-conventional type of TFT; it displays minimal neurotoxicity but contains an (RGD)-sequence at the top of the flexible loop. γ-Bungarotoxin facilitates the permeability of the vascular endothelial barrier. It binds selectively to integrin α5 in the vascular endothelium and initiates subsequent events, including dephosphorylation of focal adhesion kinase (FAK) and cytoskeletal remodeling, resulting in disruption of intercellular junctions. This promotes paracellular permeability and dysfunction of the endothelial barrier [150].

#### 4.3.7. Increased Vascular Permeability Due to Inflammation Caused by Snake Venom Toxins

Inflammation contributes to increasing vascular permeability. The local application of enzymatically active snake toxins, in particular, PLA_2_s, SVMPs, and hyaluronidases, stimulates the production and release of pro-inflammatory compounds such as arachidonate and its metabolites, prostaglandin E2, TNF-α, histamine, hyaluronan fragments, and others, which leads to additional damage to the integrity of the vascular wall and disruption of its normal functioning [83,147].

Extensive inflammation-mediated extravasation of fluid can lead to tissue edema. Inflammation-induced pulmonary hemorrhage and edema may occur as pulmonary complications of snakebites [83]. So, in a murine model, a P-III SVMP from the venom of *C. simus* induces pulmonary hemorrhage. Extravasation in the lungs occurs within 15 min after intravenous injection of the toxin, and hemorrhage intensifies after 6 h. Cleavage products of BM proteins in lung homogenates and in bronchoalveolar lavage fluid imply an enzymatic disruption of the BM at the capillary–alveolar barrier. Thus, this P-III SVMP induces acute lung injury through its direct enzymatic action on the capillary–alveolar barrier integrity by BM degradation and as a consequence of the inflammatory reaction that develops in lung tissue [151]. It should be noted that non-hemorrhagic SVMPs may also evoke a pronounced inflammatory response in human microvascular endothelial cells [152].

As can be seen from the data considered in this review, there are many toxins that have a variety of effects on the walls of blood vessels. To avoid confusion for the reader, they are summarized in tables for clarity (Table 1, Table 2 and Table 3).

## 5. Anti-Atherosclerotic Activity

The mechanisms by which some snake toxins achieve their anti-atherosclerotic effects are not always obvious. For some of them, in particular disintegrins, the anti-atherosclerotic effect is explained by their antithrombotic action [3]. In cholesterol-fed rabbits, the serine proteinase Defibrase decreases the mean aortic plaque area in the thoracic and abdominal aorta as well as the total cholesterol content compared with control animals. In addition, Defibrase treatment reduces the number of plaques at the small artery openings in the thoracic aorta and the abdominal aorta [153]. These effects of Defibrase, however, may be also related to its antithrombotic action.

As mentioned in Section 2.6 and Section 3.6, the PLA_2_ crotoxin exerts anti-inflammatory, antioxidative, and immunomodulating effects on HUVECs in vitro, along with a vasorelaxant activity. Considering that the initial stages of atherosclerosis are characterized by vasoconstriction, increased levels of adhesion molecules, inflammatory cytokines, and oxidative stress in the vascular endothelium, and that crotoxin downmodulates all these events [42], one might suggest that it has vessel-specific anti-atherogenic action in vivo.

Nicotine exposure is strongly associated with atherosclerosis. Endothelial to mesenchymal transition (EndMT) contributes to nicotine-induced atherosclerosis. Human aortic endothelial cells highly express α7nAChR; α-bungarotoxin, being an efficient α7 nAChR inhibitor, can effectively reduce nicotine-induced EndMT of these cells in vitro and atherosclerotic lesions in mice in vivo [154].

## 6. Conclusions and Prospects

The cardiovascular system, in general, and the circulatory system, in particular, are the first to be exposed to the toxic effects of snake venoms, in addition to local effects. As stated in the introduction, these effects can be quite diverse. In this review, we have tried to systematize the data on the effects of various snake toxins on the walls of blood vessels, which has not been performed so far. The data presented in the review indicate that most of the effects are unfavorable. However, a number of toxins have a positive effect on the walls of blood vessels, which may find practical application in the future.

The vast majority of snake toxins are proteins and peptides with their own advantages and disadvantages, which determine their applicability in science and practice. On the one hand, their molecules are usually larger than those of traditional pharmaceuticals, penetrate the body’s protective barriers more poorly, are usually subject to proteolysis, and are a priori immunogenic. On the other hand, they are often more selective and have higher affinity for their biological targets than low-molecular-weight compounds. The relatively large size of the protein toxin molecule allows for the introduction of reporter groups or labels without significant influence on its biological activity, which allows the molecular mechanisms of their action to be studied using sophisticated physical methods.

This review shows that snake toxins can affect the blood vessel wall in a wide variety of complex ways. In this regard, many of them can be considered fine biochemical tools—either themselves or as chemical derivatives—when studying the structures and functioning of blood vessels, as well as when modeling certain pathological conditions herein. In particular cases, these toxins have the potential to be used as prototypes of medicines or even as medicines. In general, snake toxins as biochemical tools make a significant contribution to the study of the fine molecular mechanisms of the functioning of various body systems. Almost all the groups of toxins mentioned in this review can be utilized, in a variety of ways, as tools for studying the structures and functioning of the vascular wall. In particular, disintegrins and metalloproteases are used, among others, to study the adhesion processes of endothelial cells; ion-channel blockers (proteins of the CRISP family, calciseptin) are useful for assessing the effect of ion currents on the contractility of the vascular wall. VEGF-F, alternagin-C, and other toxins mentioned in Section 3 may serve as tools in neovascularization studies. The high binding affinity of the toxin to its target in the vessel wall allows for the receptor–ligand crystal structure to be established. For example, the crystal structure for human endothelin receptor in complexes with sarafotoxins has been solved recently [20]. Snake toxins may also help to discover new properties of vascular wall components. For example, an [^125^I]-derivative of DNP from mamba venom has been used to characterize a natriuretic peptide receptor of type A (NPR-A) in human mammary arteries as well as to provide, for the first time, evidence for the receptor presence on vascular smooth muscle cells, but not endothelial cells. This finding suggests that the vasodilatation caused by DNP is predominantly mediated via direct activation of smooth muscle NPR-A [12].

Because of their small size, peptides are easier to turn into drugs than proteins. Of all the toxins discussed in this review, perhaps the most striking example are the BPPs. Using their structure as a template, a whole class of antihypertensive drugs, namely ACE inhibitors, has been created and developed. Another example is the cardiovascular drug Cenderitide, which was designed as a chimeric peptide composed of a human C-type natriuretic peptide abundantly produced in the endothelium fused with the C-terminal fragment of DNP, a natriuretic peptide from mamba venom [155].

From snake toxins, disintegrins are among the most active effectors of the vascular wall. The structures of the disintegrins echistatin and barbourin were used as the basis for the design of the drugs Tirofiban and Eptifibatide, respectively, which were approved by the FDA as antiplatelet medicines in 1998 [62]. It should be noted that echistatin and, to a much lesser extent, barbourin strongly inhibit HUVEC adhesion to fibronectin and, especially, vitronectin [66]. Although there are no clinical studies using other snake venom disintegrins to date, human ADAMs have been employed in clinical trials for the therapy of epithelial dysfunction (NCT00898859) [156], portal hypertension at cirrhosis (NCT04267406) [157], and pulmonary arterial hypertension (NCT05478226) [158].

The snake venom PLA_2_ crotoxin has passed phase I of clinical trials on patients with solid tumors, and recommended doses for phase II have been determined [159,160]. However, its application in humans is associated with certain difficulties due to the significant toxicity of this protein. To improve the safety of crotoxin, nanostructured mesoporous silica (SBA-15) has been proposed as its carrier [161]. Another way to solve this problem is to use peptides derived from the crotoxin B sequence, which are non-toxic while retaining, to some extent, the therapeutic potential of crotoxin [162].

We can speculate about the possible protective effect of snake venom toxins based on the effects of their mammalian counterparts. For instance, VEGF-A definitely has a local protective effect on blood vessels, and the drug Neovasculgen (a plasmid with VEGF-A) has been used since 2013 in Russia and Ukraine to restore blood vessels in peripheral arterial diseases. However, the medicinal properties of VEGF-F from snake venom are currently unknown. This is obviously one of the areas for future research.

We believe that further study and use of snake toxins that directly affect the vascular wall will add much valuable information about the structure and function of this important part of the circulatory system.

## Figures and Tables

**Table 1:** Effects on blood vessel walls of enzymes found in snake venoms.

Snake Toxin/A Typical Representative(s)		Main Effect(s) on Vessel Wall (and Key Target/Step of Mechanism)				
		Vascular Tone	Angiogenesis	Cytotoxicity, Cell Death	Interaction with ECM	Capillary Permeability
PLA_2_	AhV_aPA from *Agkistrodon halys pallas* or HDP-1 from *Vipera nikolskii*	Vasocontraction or vasorelaxation (by cyclooxygenase metabolites)				
	MVL-PLA_2_ from *Macrovipera lebetina* or KDR-bp from *Agkistrodon piscivorus piscivorus*		Antiangiogenic (inhibition of cell adhesion and migration through integrins α5β1 or VEGFR-2)			
	Bothropstoxin-I from *Bothrops jararacussu*			Necrosis; apoptosis (downregulation of anti-apoptotic proteins; DNA damage)		
	CC-PLA2-1 and -2 from *Cerastes cerastes*				Loss of cell adhesion (αv (αvβ3), α2, α5β1 integrins)	
SVMP	BthMP from *Bothrops moojeni*	Antiangiogenic (inhibition of cell adhesion and migration; change in gene expression)				
	VAPs (Crotalinae)		Caspase-3-dependent apoptosis (cell detachment through αvβ5, α3, α6, β1 integrins)			
	BjussuMP-II from *Bothrops jararacussu*			Impedes adhesion and promotes cell detachment (degrades various ECM proteins; cleaves the TNF-α precursor)		
	BaP1 from *Bothrops asper*				Hemorrhage per rhexis and per diapedesis (degrades the BM and adhesion proteins; interferes with collagen and integrins)	
LAAO	*Bothrops* spp.		Antiangiogenic (probably through cytotoxicity)			
	All snakes			Necrosis; caspase-mediated apoptosis by ROS		
Hyaluronidase	All snakes				Digests hyaluronan	Increases

**Table 2:** Effects on blood vessel walls of non-enzymatic proteins of snake venoms.

Snake Toxin (Source Venom)		Main Effect(s) on Vessel Wall (and Key Target/Step of Mechanism)					
		Vascular Tone	Angiogenesis	Cytotoxicity, Cell Death	Interaction with ECM	Capillary Permeability	Anti-Atherosclerotic
VEGF-F (Viperidae)		Vasorelaxation (increases NO production)	Promotes endothelial cell proliferation and migration (VEGFR-2)			Increases (VEGFR-1 and VEGFR-2)	
CRISP	(Australian and sea Elapidae)	Vasocontraction (BKCa) or Vasorelaxation (L-type Ca^2+^ channel)					
	ES-CRISP from *Echis carinatus sochureki*		Antiangiogenic (growth factor signaling)				
	(Crotalinae)					Increases (through different pathways)	
C-type lectins and snaclecs (Viperidae)			Antiangiogenic (cell adhesion and migration inhibition: integrins αvβ3, αvβ5, and α5β1, and VEGF signaling)				
Disintegrin (Viperidae)		Inhibits angiospasm at thromboembolism (reduces the adhesion of platelets to the subendothelium)	Inhibition (integrins αvβ3, α5β1, α1β1, and α2β1) Promotion (growth factor signaling)	Caspase-3-dependent apoptosis (αvβ5 integrin; DNA fragmentation)	Detaches and rounds up cells (integrins vβ3 or α1β1)	Prevents vascular leakage (integrin αvβ3; VEGF signaling)	
TFT	Cytotoxins (*Naja* genus)	Vasocontraction (Ca^2+^ channel)		Both necrotic and apoptotic (lysosomal damage, violation of energy metabolism)			
	Muscarinic toxins MTα and MT9 from *Dendroaspis polylepis*	Vasorelaxation (α2B adrenoreceptor or M2 muscarinic AChR)					
	α-Bungarotoxin from *Bungarus multicinctus*		Inhibits nicotine-evoked endothelial network (abrogates upregulation of VEGF and bFGF gene expression)				Reduce atherosclerotic lesions (nicotine-induced EndMT)
	γ-Bungarotoxin from *Bungarus multicinctus*				Promotes (integrin α5)		
	Calciseptin and FS2 from *Dendroaspis polylepis*	Blocks contraction (L-type Ca^2+^ channels)					
Kunitz-type ISP from *Macrovipera lebetina transmediterranea*			Antiangiogenic (inhibits cell adhesion and migration; integrin αvβ5)				

**Table 3:** Effects on blood vessel walls of peptides and low-molecular-weight components of snake venoms.

Snake Toxin		Source Venoms	Main Effect on Vascular Tone (and Key Target/Step of Mechanism)
Peptides	BPP	*Bothrops* spp.	Vasorelaxation (Releases NO from the endothelium)
	NP	Different genera of Viperidae and Elapidae families	Vasorelaxation (Increases NO production; K^+^ channels)
	Sarafotoxins	*Atractaspis* spp.	Vasoconstriction or vasorelaxation (ET-A and ET-B receptor)
Low-MW	Adenosine	All snakes	Vasorelaxation (adenosine A2 receptors)

## Data Availability

Not applicable.

## Abbreviations

The following abbreviations are used in this manuscript:

ACE1	Angiotensin-converting enzyme 1
ADAM	A disintegrin and metalloproteinase
AR	Aortic ring
AsS	Argininosuccinate synthase
BCE	Bovine capillary endothelial
bFGF	Basic fibroblast growth factor
BM	Basement membrane
BKB2	Bradykinin receptor B2
BPP	Bradykinin-potentiating peptide
CAM	Chorioallantoic membrane
CRISP	Cysteine-rich secretory protein
CLLP	C-type lectin-like protein, or Snaclec
CTL	C-type lectin
CTX	Cytotoxin
ECM	Extracellular matrix
ET	Endothelin
FAK	Focal adhesion kinase
HBMEC	Human brain microvascular endothelial cell
HDMEC	Human dermal microvascular endothelial cell
HMEC-1	Human microvascular endothelial cell line-1
HUVEC	Human umbilical vein endothelial cell
ICPP	Increasing capillary permeability protein
MDLA	Methyl-D-L-aspartic acid
NP	Natriuretic peptide
NPR	Natriuretic peptide receptor
LAAO	L-amino acid oxidase
L-NAME	N(ω)-nitro-L-arginine methyl ester
NA	Noradrenaline
nAChR	Nicotinic acetyl choline receptor
NGF	Nerve growth factor
NOS	NO-synthase
PLA_2_	Phospholipase A_2_
ROS	Reactive oxygen species
SVMP	Snake venom metalloproteinase
TFT	Three-finger toxin
VAP	Vascular apoptosis-inducing protein
VEGF	Vascular endothelial growth factor
VEGFR	VEGF Receptor
